# Lattice-engineered site symmetry control of Bi^3^⁺ activators for tunable luminescence and latent fingerprint detection

**DOI:** 10.1038/s41598-026-47106-4

**Published:** 2026-04-08

**Authors:** Aditya T. Jagdale, Praful P. Khode, Gaurav V. Mishra, Tushar R. Shelke, Naumov G. Nikolay, S. J. Dhoble

**Affiliations:** 1 Department of Physics, R.T.M. Nagpur University, Nagpur, 440033 India; 2 Datta Meghe Institute of Higher Education & Research, Sawangi (Meghe), Wardha, 442001 India; 3 Department of Applied Physics, K.D.K. College of Engineering, Nagpur, India; 4 Institute of Inorganic Chemistry, Siberian Branch of the Russian Academy of Science, 630090 Novosibirsk, Russia

**Keywords:** Materials science, Optics and photonics, Physics

## Abstract

Optical functionality in luminous materials can be effectively tailored by manipulating the local symmetry environment of activator ions. To study the impacts of lattice engineering on photoluminescence and latent fingerprint detection, urea-assisted combustion was used to create Bi^3^⁺-doped Y₂O₃ phosphors co-doped with alkali metal ions (Li⁺, Na⁺, K⁺, Cs⁺). Prepared Y₂O₃ phosphors with co-doped with alkali metal ions has cubic phase with space group I –a 3 with dopant-induced lattice contraction and smaller crystallites. In FTIR study we observed vibrational bond due to Y-O stretching. Prepared phosphor has irregular particles size with average particle size ~140 nm. Bi^3+^ ions occupy C₂ and S₆ symmetry positions, according to optical investigations, resulting in separate ^1^S₀→^3^P₁ and ^1^S₀→^1^P₁ transitions. Alkali co-doping increased intensity without changing the spectrum, however emission changed from bluish-white (329–337 nm excitation) to blue (374 nm). Y₂O₃: Bi/Na demonstrated exceptional fingerprint visualization and nearly 100% color purity. These findings demonstrate that tunable luminescence for sophisticated photonic and forensic applications is made possible by Bi^3⁺^ site-symmetry modification via alkali lattice engineering.

**Supplementary Information:**

The online version contains supplementary material available at 10.1038/s41598-026-47106-4.

## Introduction

The development of efficient anti-counterfeiting techniques is crucial since the risk of authenticity has increased due to the rise in luxurious lifestyles . Counterfeiting not only causes financial loss but also a safety concerns so^1,2^, The identification of individuals by distinctive fingerprint patterns continues to be one of the most precise, trustworthy, and legally recognized forms of personal authentication in the age of quickly developing forensic technology. However, latent fingerprints—those that are invisible to the unaided eye—on intricate or shiny surfaces frequently provide serious difficulties for conventional optical and photographic methods^3,4^.

In order to get over these restrictions, scientists are concentrating more on creating luminescent materials that, when excited by ultraviolet light, can produce powerful and adjustable light. By converting UV radiation into visible emission, these phosphor-based materials make it possible to see fingerprint ridge features in high contrast with improved sensitivity and selectivity. Among them, rare-earth and transition-metal-doped oxide phosphors have garnered increasing interest due to their effective photoluminescence, chemical stability, and environmental durability, which make them perfect options for next-generation forensic detection systems^5,6^.

In recent years, there has been a significant rise in interest in luminescent materials because of their increasing use in energy-efficient lighting, radiation detection, anti-counterfeiting measures, biomedical imaging, and display technologies. Specifically, researchers are concentrating on creating phosphors that emit visible light when exposed to UV or near-UV light, which are crucial for contemporary white light-emitting diode (WLED) devices. The pursuit of innovative phosphor compositions exhibiting high emission efficiency, thermal stability, and tunability in their spectral output has resulted in considerable research involving host materials doped with rare-earth and transition metals. Ongoing trends focus on optimizing synthesis techniques to manage morphology, minimizing concentration quenching, and modifying the local crystal field environment to improve radiative transitions^7–10^.

Among the various host materials, yttrium oxide (Y₂O₃) is notable for its remarkable optical characteristics, chemical endurance, high melting temperature, and capacity to accommodate a diverse array of dopant ions. It features a stable cubic structure along with a broad bandgap, rendering it appropriate for studies in both photoluminescence (PL) and thermoluminescence (TL). Additionally, Y₂O₃ demonstrates low phonon energy and reduced non-radiative losses, facilitating effective energy transfer between the host lattice and the dopant ions. It has been widely utilized in phosphor-converted LEDs (pc-WLEDs) and thermoluminescent dosimeters, making it an adaptable platform for luminescence engineering ^11–14^. Dopant ions like Bi^3+^ or rare earth ions can occupy various local habitats because Y₂O₃ has a cubic structure with three different cationic sites within its host lattice. A wide and intricate luminescence profile is produced as a result of the inhomogeneous broadening of the emission bands caused by this site variation^15,16^.

Incorporating bismuth (Bi^3+^) into Y₂O₃ results in broad-band emissions in the visible spectrum due to allowed transitions from 6s^2^ to 6s6p. The ionic size of Bi^3+^ is similar to that of Y^3+^, facilitating its substitution in the Y₂O₃ crystal structure. Bi^3+^ ions occupy two different crystallographic positions (S₆ and C₂), which typically produce emissions around ~408 nm and ~505 nm. These ions play a role in modulating the local environment, enhancing charge compensation, diminishing non-radiative centres, and causing lattice distortion, all of which work together to increase emission intensity^17–19^.

Prior research has explored the synthesis and luminescent properties of Bi-doped Y₂O₃ through various approaches, including solid-state and combustion methods. Jacobsohn et al. observed the dual emission characteristics of Bi^3+^ in nanocrystalline Y₂O₃ and emphasized how the synthesis conditions affect the occupancy of dopant sites^20^. Dutta et al. revealed how co-doping with rare-earth and alkali ions influences energy transfer and lifetime characteristics, indicating substantial enhancements in emission efficiency^21^. Nonetheless, there is still a gap in the literature regarding the thorough optimization of alkali co-doping in Y₂O₃: Bi systems for photoluminescence performance.

In this study, we seek to address these limitations by producing Bi-doped Y₂O₃ nanophosphors through a solution combustion technique that uses urea as the fuel, and we further improve their luminescent properties by co-doping with alkali metal ions (Li^+^, Na^+^, K^+^, Cs^+^). The synthesized phosphors undergo analysis for their structural analysis using XRD (X-ray diffraction), morphology by SEM (Scanning electron microscopy), bond structure using Fourier Transform Infrared spectroscopy (FT-IR) and photoluminescence (PL) characteristics. Our findings indicate that alkali co-doping leads to increased emission intensity and enhanced thermal stability, highlighting their potential for practical applications such as blue-emitting phosphors in white LEDs and fingerprint detection technologies.

## Synthesis

**Fig. 1:**
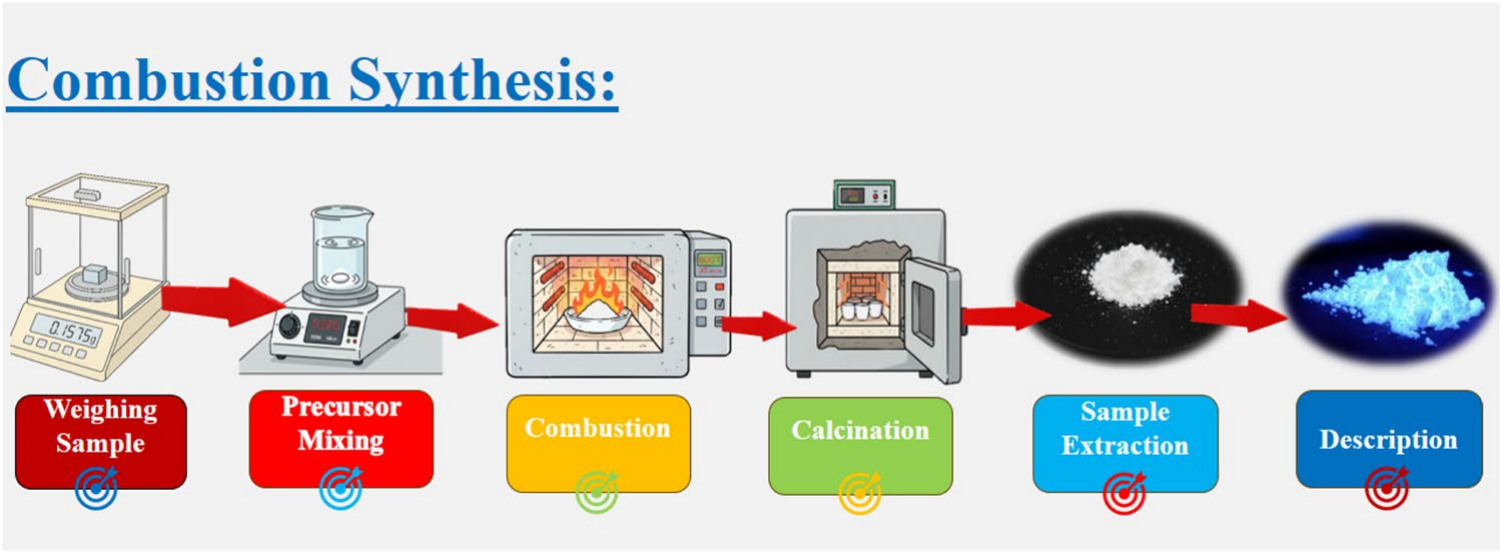
Synthesis diagram of solution combustion synthesis.

In this research, Y₂O₃: Bi was produced through the solution combustion synthesis (SCS) technique. A specified amount of Y(NO₃)₃·6H₂O was placed into five different 100 mL beakers and labelled with 1-5 numbers, dissolved in distilled water, and stirred until complete homogeneity was achieved. Separately, the necessary quantity of bismuth subcarbonate was measured into five test tubes (label them 1-5) and dissolved in a small volume of nitric acid. After stirring for 15–20 minutes, the bismuth solution was combined with the yttrium nitrate solution in the beaker, followed by an additional stirring period of 15–20 minutes to ensure thorough mixing. Afterward, 4 g of urea, which acted as the fuel for the combustion process, was incorporated into the mixture and stirred for another 15–20 minutes until it was completely dissolved. The resulting solution was then transferred to a china dish and placed in a furnace set at 550 °C. Combustion began, as indicated by the emergence of red flames, transforming the liquid precursor into a powder. The obtained powder was grind using a mortar and pestle, placed into a crucible sequentially, and calcined at 800 °C overnight. The sample was permitted to cool naturally to room temperature, grind again to achieve fine particles, and stored in a sample bottle for future characterization. To enhance the properties, the powders were additionally doped with alkali elements (Li, Na, K, Cs). This doping took place before the urea addition. The calculated quantities of alkali salts were dissolved in nitric acid and added to the beaker after the bismuth solution, ensuring a 15–20 minute interval of stirring before continuing with the subsequent mixing processes of urea and followed the same process of combustion in a china dish and calcination. Figure. 1 shows the synthesis route of solution combustion.

## Results and discussion

### XRD

**Fig. 2:**
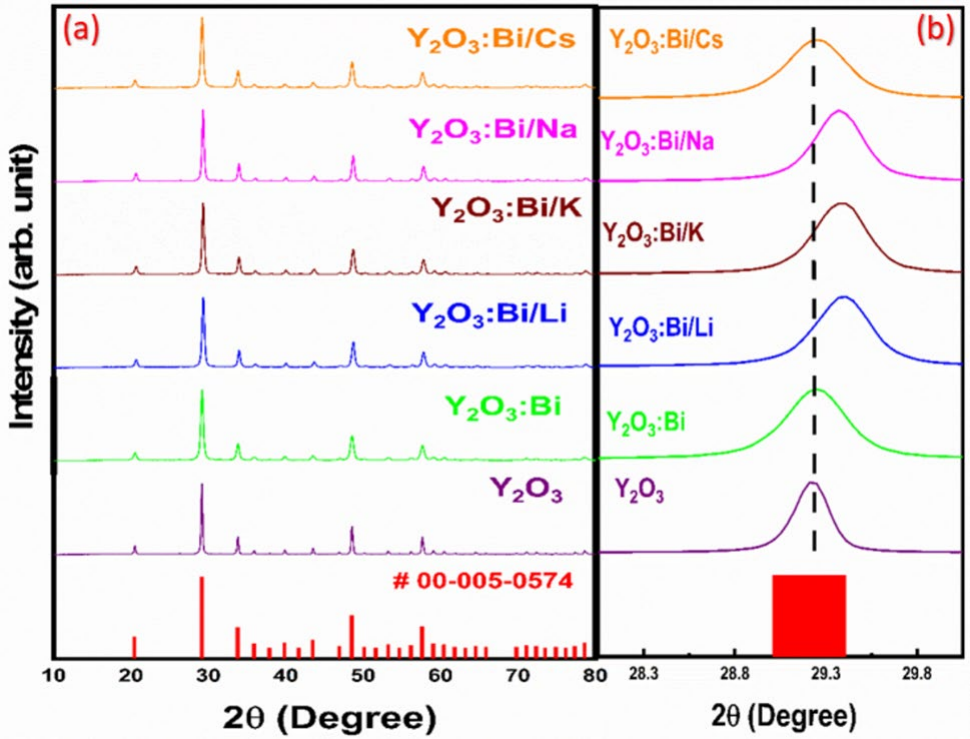
XRD pattern of Y₂O₃, Y₂O₃: Bi , Y₂O₃: Bi/Li , Y₂O₃: Bi/Na , Y₂O₃: Bi/K,and Y₂O₃: Bi/Cs with the standard data.

XRD pattern of the prepared phosphor of Y₂O₃, Y₂O₃: Bi(0.3 mol%) , Y₂O₃: Bi(0.3 mol%)/Li(0.9 mol%), Y₂O₃: Bi(0.3 mol%)/Na(0.9 mol%), Y₂O₃: Bi(0.3 mol%)/K(0.9 mol%), and Y₂O₃: Bi(0.3 mol%)/Cs(0.6 mol%)with standard data is shown in Fig. 2(a). XRD spectrum is recorded from 10° to 80° with a step size of 0.04°. ICSD Card No. of standard data is 00-005-0574. When we compare all prepared phosphors with standard data, all major and minor peaks of the XRD pattern are matched with standard data. This confirm the formation of Y₂O₃. When we add dopants like Bi(bismuth), K(potassium), Na(sodium), Cs(cesium), and Li(lithium) to the host ion with very little concentration of host during synthesis. Selecting that concentration for XRD analysis is due to it having the maximum PL emission intensity in its corresponding series of samples. On comparing the standard data of Bi_2_O_3_, K_2_O, Na_2_O, Li_2_O, and Cs_2_O with the experimental option result of the above phosphor. There is no such peak present in the above XRD pattern of the prepared phosphor, which confirms that no other phase is present due to the dopant in the host lattice. Dopant is successfully incorporated in the host lattice with a shift in the XRD pattern compared with the undoped Y₂O₃. All prepared phosphor is single-phase phosphor due to the absence of other peaks. Sharp line in XRD pattern confirms the high crystallinity of the prepared phosphor.

Full Width Half Maxima (FWHM) of peak between 28 ^o^ to 29.5° of Y₂O₃, Y₂O₃: Bi(0.3 mol%) , Y₂O₃: Bi(0.3 mol%)/Li (0.9 mol%), Y₂O₃: Bi(0.3 mol%)/Na(0.9 mol%) ,Y₂O₃: Bi(0.3 mol%)/K(0.9 mol%),and Y₂O₃: Bi(0.3 mol%)/Cs(0.6 mol%) is shown in the Table 1. Table 2 contain the ionic radius of ion such as Y, Bi, Na, Li, K, and Cs. The alkali co-doped samples show a minor shift of diffraction peaks toward higher 2θ values in the following order: Y₂O₃ ≈ Y₂O₃: Bi ≈ Y₂O₃: Bi/Cs < Y₂O₃: Bi/Na < Y₂O₃: Bi/K < Y₂O₃: Bi/Li. Bragg’s Law states that a small lattice contraction is indicated by this right shift, which is equivalent to a decrease in interplanar spacing. The different ionic radii and substitution behavior of the alkali ions in the Y₂O₃ lattice are responsible for this behavior. While larger ions like Cs^+^ have a lower chance of replacing Y^3+^ sites and are more likely to occupy interstitial positions or segregate at grain boundaries, leading to negligible peak shift, smaller ions like Li^+^ can more easily occupy lattice sites and cause stronger local contraction of the lattice. On the other hand, the diffraction peaks’ full width at half maximum (FWHM) increases regularly in the following order: Y₂O₃ < Y₂O₃: Bi/Na < Y₂O₃: Bi/K < Y₂O₃: Bi/Li < Y₂O₃: Bi < Y₂O₃: Bi/Cs. Lattice strain, defect development, and the reduction in crystallite size brought about by dopant inclusion all contribute to this peak broadening. Due to variations in ionic size and electronic configuration, the presence of Bi^3^⁺ ions can cause local structural distortion, whereas the addition of monovalent alkali ions creates a charge imbalance that is offset by the creation of oxygen vacancies and other point defects. The Scherrer Equation states that a decrease in crystallite size and an increase in lattice microstrain are correlated with an increase in FWHM. The very large ionic radius of Cs^+^, which substantially inhibits crystallite formation by promoting grain boundary segregation and significant structural disorder, is responsible for the exceptionally large FWHM seen in the Cs co-doped sample. Consequently, the variation in FWHM is predominantly controlled by crystallite size reduction, microstrain, and defect density, including oxygen vacancies produced during the co-doping process, whereas the peak shift mostly reflects variations in lattice spacing induced by ionic size mismatch.

**Table 1:** FWHM of Y₂O₃, Y₂O₃: Bi , Y₂O₃: Bi/Li , Y₂O₃: Bi/Na , Y₂O₃: Bi/K, and Y₂O₃: Bi/Cs.

FWHM of sharp peak					
Y₂O₃	Y₂O₃: Bi	Y₂O₃: Bi/Li	Y₂O₃: Bi/K	Y₂O₃: Bi/Na	Y₂O₃: Bi/Cs
0.2011 ^o^	0.386 ^o^	0.345 ^o^	0.328 ^o^	0.302 ^o^	0.386 ^o^

**Table 2:** Ionic radii of Y, Bi, Li, K, Na and Cs.

Ionic radii of ion					
Y	Bi	Li	K	Na	Cs
1.019 Å ^22^	1.17 Å ^22^	0.92 Å ^22^	1.12 Å ^22^	1.51 Å^22^	1.74 Å ^22^

Crystalline Size of Y₂O₃, Y₂O₃: Bi , Y₂O₃: Bi/Li , Y₂O₃: Bi/Na , Y₂O₃: Bi/K, and Y₂O₃: Bi/Cs is shown in the Table 3. Adding a dopant to the host lattice reduces the crystalline size of the prepared phosphor. So Y has crystalline equal to 36.09, which is greater than other doped phosphors. Y₂O₃ grains can expand to a bigger size through processes like Ostwald ripening because crystal development happens with reasonably consistent kinetics in the absence of dopants. When dope Bi ion in host lattice crystalline size is smallest among all phosphors, due to Bismuth doping acts as a potent grain growth inhibitor by pinning boundaries with induced strain, severely reducing crystallite size. Adding alkali earth metal increases the crystalline size due to complex effects introduced by the addition of aliovalent alkali ions (Li⁺, Na⁺, K⁺, and Cs⁺), which are bigger and have a lower charge than Y^3^⁺. The defect landscape is changed by their inclusion, which might happen interstitially or necessitate charge-compensating defects like oxygen vacancies. While some alkali compounds can create a brief liquid phase during calcination that functions as a flux to improve atomic diffusion and encourage sintering, this may partially offset the strain from Bi, lessening its pinning effect. In the end, compared to the Bi-only doped sample, this intricate interaction of changing strain, liquid-phase sintering, and changed defect chemistry promotes larger crystallite development. On comparing the crystalline size between alkaline earth metals. The distinct effectiveness of each alkali co-dopant in modifying crystallite size stems from a competition between its ionic size and its influence on the material’s sintering behaviour. Because of its reasonable size, which optimally relieves Bismuth’s lattice strain without excessive segregation and may produce a low-melting eutectic that serves as a very effective flux for atomic diffusion, sodium (Na) proved to be the most successful at encouraging growth. The larger potassium (K) and smaller lithium (Li) ions, on the other hand, were less effective, maybe because K starts to separate at boundaries and Li generates distinct lattice distortions, both of which partially reintroduce growth obstacles. Notably, Cesium (Cs), the largest ion, exhibited the weakest promoting effect because of its extreme size, which probably causes severe segregation at grain boundaries, effectively pinning them and thus most strongly counteracting the growth-promoting mechanisms. This results in crystallites that are closest in size to the Bi-only sample.

**Table 3:** Crystalline size of Y₂O₃, Y₂O₃: Bi , Y₂O₃: Bi/Li , Y₂O₃: Bi/Na , Y₂O₃: Bi/K, and Y₂O₃: Bi/Cs.

Crystalline size using the Debye–Scherrer method					
Y₂O₃	Y₂O₃: Bi	Y₂O₃: Bi/Li	Y₂O₃: Bi/K	Y₂O₃: Bi/Na	Y₂O₃: Bi/Cs
36.09	19.86	24.46	25.53	27.70	21.60

**Fig. 3:**
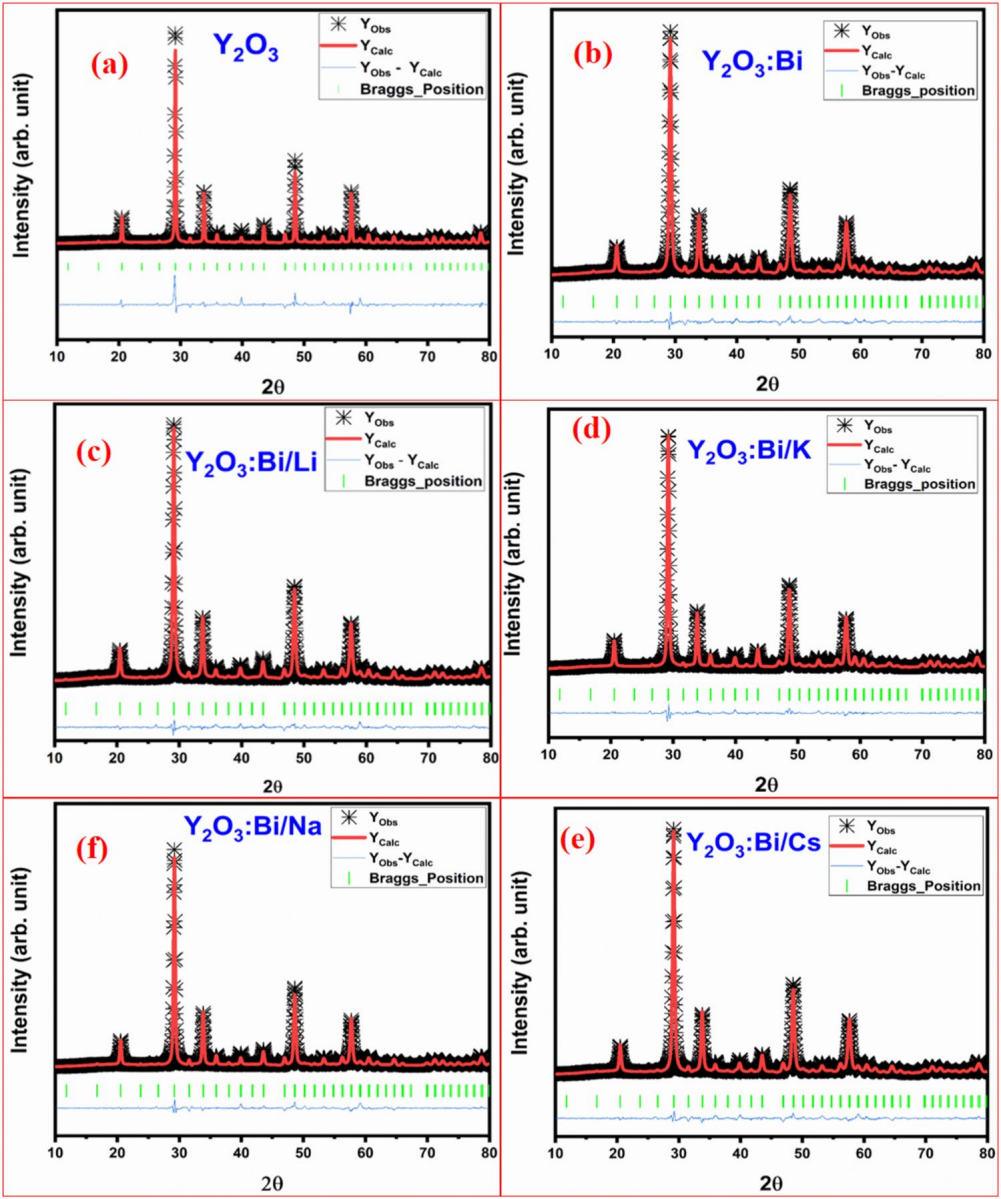
Rietveld refinement of Y₂O₃, Y₂O₃: Bi , Y₂O₃: Bi/Li , Y₂O₃: Bi/Na , Y₂O₃: Bi/K, and Y₂O₃: Bi/Cs.

Prepared phosphor Y₂O₃_,_ its co-doped and doped samples have a cubic crystal structure. Prepared phosphor has I –a 3 space group^23^. The I –a 3 space group characterizes a non-centrosymmetric body-centred cubic lattice with particular screw axes^14^. It is distinguished by translations along the axes and 180° rotations. Y₂O₃ (bixbyite structure) adopts this particular cubic crystal form, in which the atomic arrangement is devoid of inversion symmetry. In the lattice, there are 3 different cryptographically distinct sites of the Y^3+^ ion named as Y1, Y2, and Y3. There are two different O^2-^ sites present in the host lattice. The crystal structure is shown in Fig. 3.

**Fig. 4:**
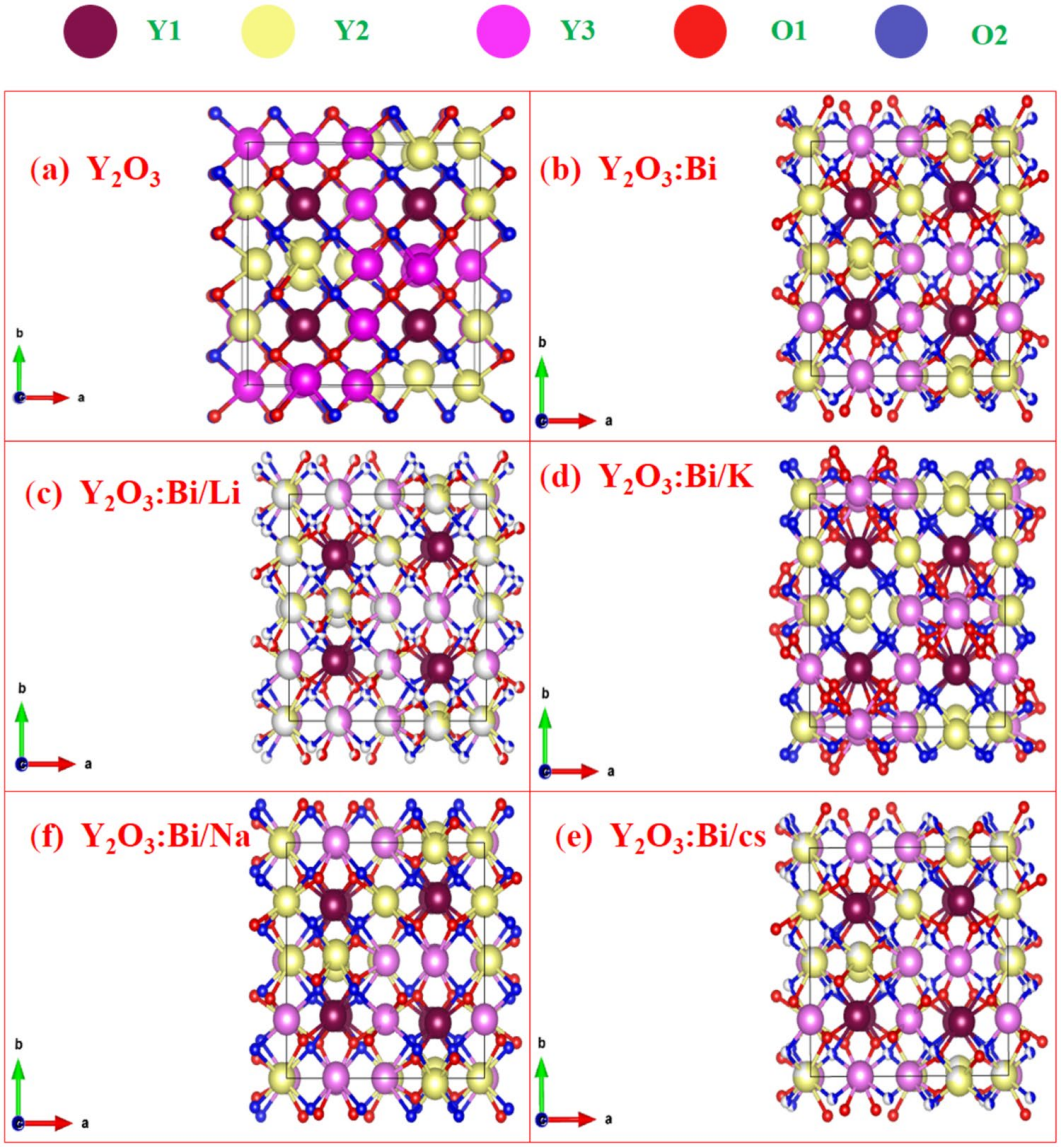
Crystal structure of Y₂O₃, Y₂O₃: Bi , Y₂O₃: Bi/Li , Y₂O₃: Bi/Na , Y₂O₃: Bi/K, and Y₂O₃: Bi/Cs.

The effect of doping on the lattice parameter of the prepared phosphor is examined by using Rietveld refinement of the prepared phosphor. Rietveld refinement of Y₂O₃, Y₂O₃: Bi, Y₂O₃: Bi/Li, Y₂O₃: Bi/Na, Y₂O₃: Bi/K, and Y₂O₃: Bi/Cs is shown in Fig. 4. The parameter that determines the quality of refinement is shown in Table 4. The ionic radii of the dopant ions, charge compensation effects, and structural distortions produced within the host lattice can all be used to explain the variance in lattice parameter values among the doped samples. Although Bi^3^⁺ has a greater ionic radius (1.03 Å) than Y^3^⁺ (0.90 Å), a minor drop in lattice parameter is found in the Bi-doped sample (Y₂O₃: Bi), which may be caused by local lattice distortion and increased Bi–O bond formation. The lattice expands significantly when Li⁺ and Bi^3^⁺ (Y₂O₃: Bi/Li) are co-doped because the smaller Li⁺ ion (0.76 Å) and the charge difference produce oxygen vacancies to preserve charge neutrality. The lattice parameter noticeably increases as a result of these vacancies, weakening the overall lattice bonding. The ionic radius of Na⁺ (1.02 Å) in the Bi + Na co-doped sample (Y₂O₃: Bi/Na) is similar to that of Bi^3^⁺, resulting in a highly stable structure with only minor contraction. The lattice exhibits contraction in Bi + K (Y₂O₃: Bi/K), despite the fact that K⁺ (1.38 Å) is significantly larger. This could be because of strain-induced tightness and structural rearrangement to account for the size mismatch. On the other hand, because the big Cs⁺ ion (1.67 Å) causes lattice stretching and increases the unit cell volume, the Bi + Cs co-doped sample (Y₂O₃: Bi/Cs) exhibits a slight expansion in lattice parameter. Overall, the interaction of ionic size difference, charge imbalance, and defect development within the crystal structure is responsible for the observed discrepancies. Table 5 contains the detailed values of lattice parameter a, α, and volume of the prepared phosphor.

**Table 4:** Rietveld refinement parameter of Y₂O₃, Y₂O₃: Bi , Y₂O₃: Bi/Li , Y₂O₃: Bi/Na , Y₂O₃: Bi/K , and Y₂O₃: Bi/Cs.

Rietveld refinement parameter					
Sr no	Name	R_p_	R_wp_	R_exp_	Chi^2^
1	Y₂O₃	9.8	12.78	7.02	3.33
2	Y₂O₃: Bi	11	11.9	7.33	2.65
3	Y₂O₃: Bi/Li	11.4	12.4	7.42	2.795
4	Y₂O₃: Bi/K	11.4	11.1	7.64	2.11
5	Y₂O₃: Bi/Na	12.8	14.5	7.11	4.13
6	Y₂O₃: Bi/Cs	11.3	12.2	7.27	2.82

**Table 5:** Lattice parameter of Y₂O₃, Y₂O₃: Bi , Y₂O₃: Bi/Li , Y₂O₃: Bi/Na , Y₂O₃: Bi/K, and Y₂O₃: Bi/Cs.

Lattice parameter							
	Y₂O₃	Y₂O₃: Bi	Y₂O₃: Bi/Li	Y₂O₃: Bi/Na	Y₂O₃: Bi/K	Y₂O₃: Bi/Cs	Standard^23^
a	10.608333 Å	10.591308 Å	10.621644 Å	10.593016 Å	10.589094 Å	10.608594 Å	10.601 Å
Δa (Å)	+ 0.007	-0.010	+ 0.021	-0.008	-0.012	+0.008	-
α	90^o^	90 ^o^	90 ^o^	90 ^o^	90 ^o^	90 ^o^	90 ^o^
Volume	1193.827 Å^3^	1188.089 Å^3^	1198.327 Å^3^	1188.663 Å^3^	1187.344 Å^3^	1193.915 Å^3^	1191.37 Å^3^

### SEM

**Fig. 5:**
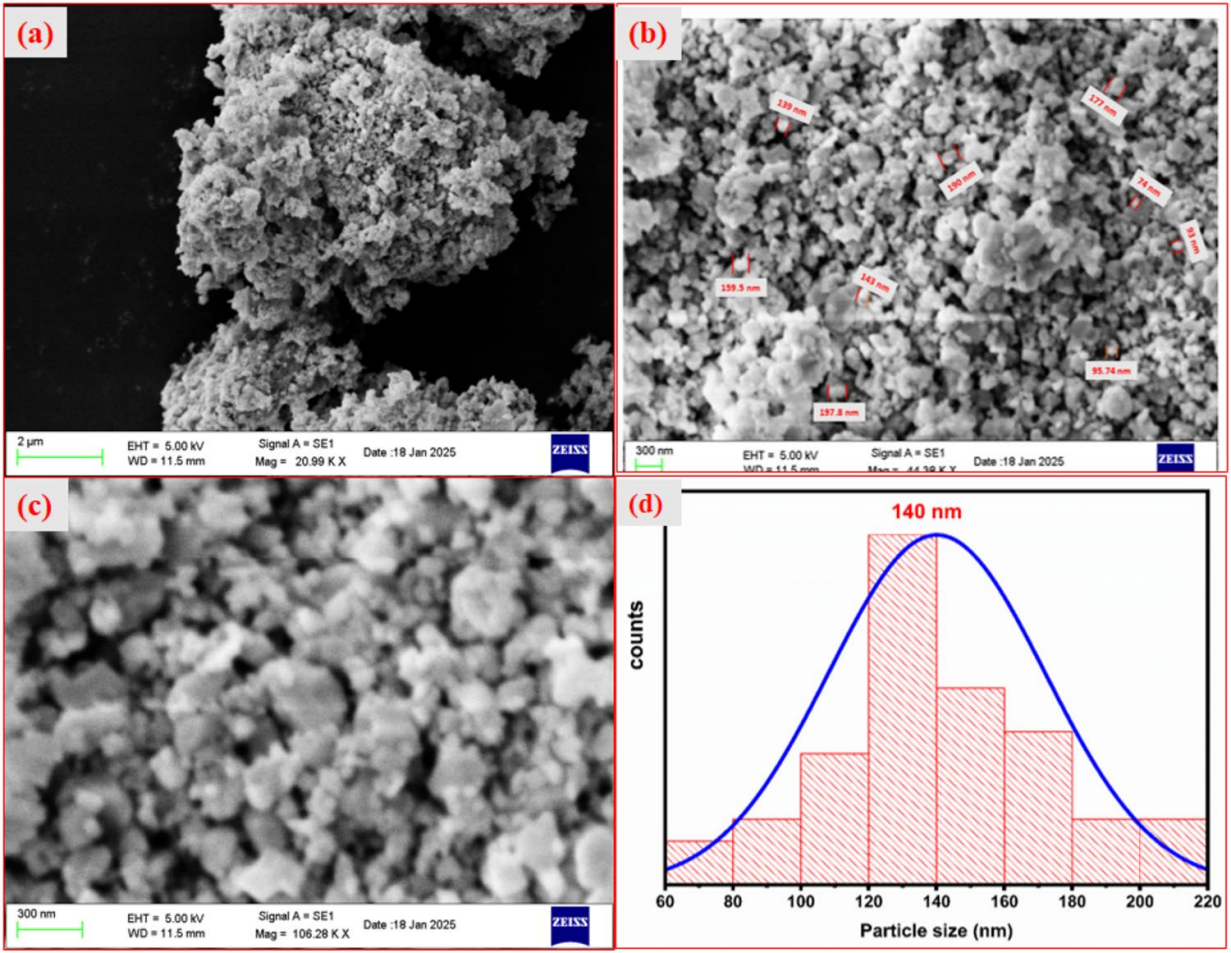
SEM image of Y₂O₃ and particle size distribution curve.

Figure 5(a), (b), and (c) show the SEM images of Y₂O₃ at 2 micrometers, 300 nm, and 300 nm, respectively. The particle is porous in nature due to the release of gases during the process of combustion. The particle is an agglomerate due to the uncontrolled increase in temperature during the process of combustion and sintering process. The particle seems to be in the micrometer range due to agglomeration. Some particles seem in the nanoparticle range, as shown in Fig. 5(b). The shape of particles is irregular in size due to the synthesis process. Figure 5(d) shows the particle size distribution curve of the prepared phosphor. We mainly focus on nanoparticles. Particle sizes are in the range from 60 nm to 220 nm with an average particle size equal to 140 nm.

### FT-IR

**Fig. 6:**
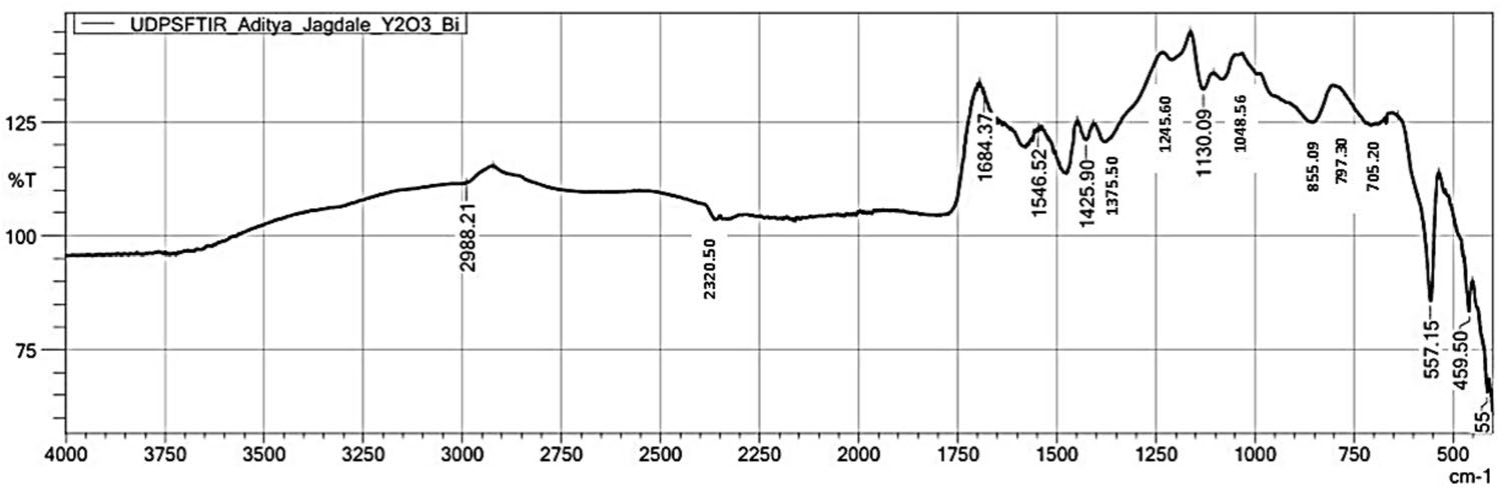
FT-IR spectrum of Y₂O₃ recorded from 400 cm^-1^ to 4000 cm^-1^.

FT-IR spectrum contains peaks at 459.50 cm^-1^ and 557.15 cm^-1^ due Y-O vibration present in the host^24,25^ as shown in Fig. 6. The Y-O bond present in the host lattice confirms the formation of Y₂O₃. Minor peak is absorbed between 1750 cm^-1^ to 1000 cm^-1^ is due to vibration of C-H or NO₃⁻. Peak at 2988.21 cm^-1^ is due to C-O vibration that confirms a small amount of C-O bond present in the host lattice^26,27^. In the above spectrum, there is an absence of a peak around 3000 cm^-1^ to 3500 cm^-1^, which confirms that the prepared phosphor does not absorb the moisture from the atmosphere. That confirms that prepared phosphor does not have a tendency to catch the moisture and turn the oxides into a hydroxide compound^28^.

### PL

#### Y₂O₃: Bi

**Fig. 7:**
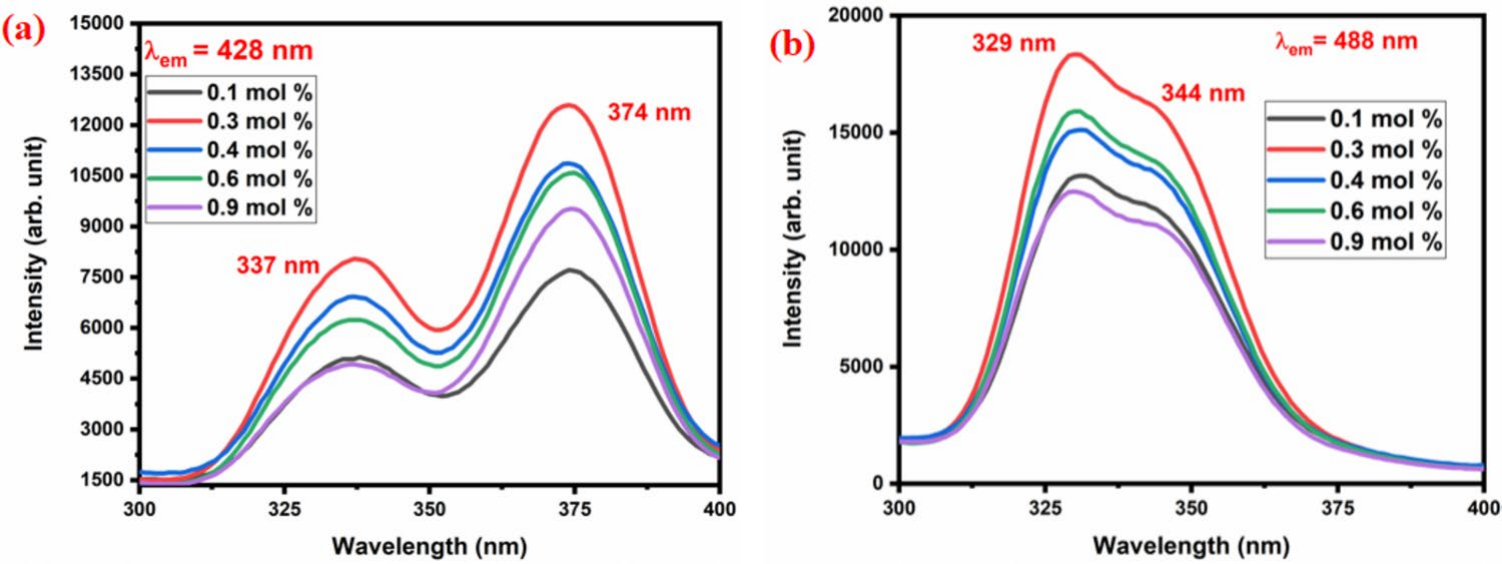
Excitation spectra of Y₂O₃: Bi at emission wavelengths 428 nm and 488 nm.

Figure 7 (a) shows the excitation spectra of Y₂O₃: Bi at emission wavelength 428 nm recorded from 300 nm to 400 nm. Excitation spectra contain two doublet board peaks, ranging from 315 nm to 400 nm. One minor peak ranges from 315 nm to 350 nm, centred at 337 nm another major peak at 374 nm ranges from 350 nm to 400 nm. The electronic configuration of the Bi^3^⁺ ion allows the transition from ground state 6s^2^ to higher state 6s^1^6p^1^. In the excitation state, possible level are ^3^P_0_, ^3^P_1_, ^3^P_2_, and ^1^P_1_. These peaks represent the intra-configurational transitions of Bi^3^⁺ ions from the excited states ^3^P_1_and ^3^P_1_ to the ground state ^1^S_0_^25,29^. While the 374 nm band ^1^S_0_ →^3^P_1_ is spin-forbidden but partially allowed because of spin–orbit coupling, the 337 nm transition ^1^S_0_ →^1^P_1_ is spin-allowed and therefore more intense. Bi^3^⁺ ions occupying both C₂ (non-centrosymmetric) and C_3i_ (centrosymmetric) sites in the cubic Y₂O₃ lattice (space group Ia-3) cause these transitions, which result in small energy changes and the development of two distinct excitation peaks that cause the blue emission at 428 nm^30,31^. Figure 7(b) shows the excitation spectra of Y₂O₃: Bi at emission wavelength 488 nm, ranges from 300 nm to 400 nm. Excitation spectra contain one broad spectral range from 315 nm to 375 nm with humped at 2 different wavelengths. One major humped at the wavelength 329 nm, while a comparable small humped at the excitation wavelength 344 nm, which are attributed to the spin-allowed ^1^S₀ → ^1^P₁ transition of Bi^3^⁺ ions occupying the cubic lattice’s two nonequivalent cation positions (C_2_ and C_3i_). The two observed humps result from a slight energy separation of the ^1^P₁ state caused by the distinct local crystal fields at these sites, whereas the strong electron-phonon coupling intrinsic to the 6s^2^ configuration and spectral overlap with the wider, underlying O^2^⁻ → Bi^3^⁺ charge-transfer band in this region and 3 different cryptographic different site present in host lattice of Y^3+^ for replacement of Bi ion are responsible for the significant spectral breadth^29^.

**Fig. 8:**
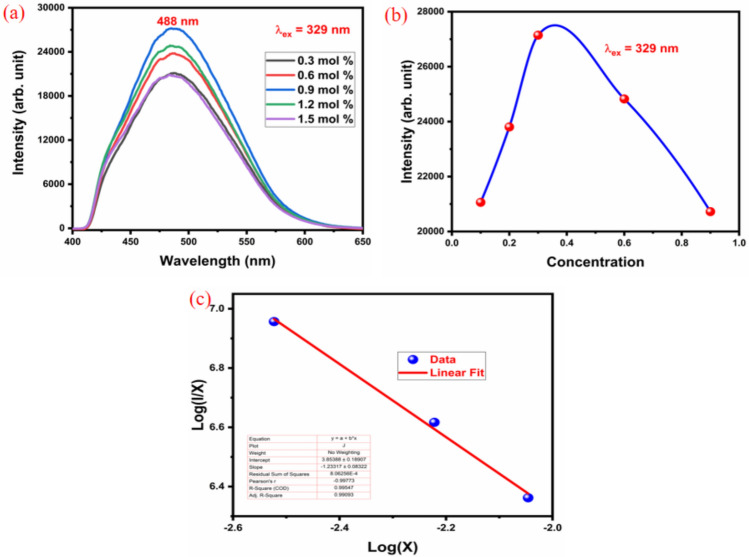
Emission spectra, concentration quenching, and Log(I/X) vs Log(X) of Y₂O₃: Bi at excitation wavelength 329 nm.

Emission spectra of Y₂O₃: Bi at excitation wavelength is shown in Fig. 8(a) at excitation wavelength 329 nm. Emission spectra are recorded in the range from 400 nm to 650 nm. Emission spectra are recorded at different concentrations of Bi ion, such as 0.1, 0.3, 0.4, 0.6, and 0.9 mol%. Emission spectra contain a broad spectrum from 415 nm to 625 nm centred at 488 nm. Variation of the concentration of the Bi ion does not change the behaviour of the emission spectra; it only changes the intensity of the emission spectra. When excited at 329 nm, Bi^3^⁺ in Y₂O₃ undergoes a ^1^S_0_→^1^P_1_ intra-configurational transition, often with overlap from an O^2^⁻→Bi^3^⁺ charge-transfer band. The triplet manifold is populated by rapid non-radiative relaxation and spin-orbit mixing, and the relaxed excited states decay radiatively, producing a broad emission from ~400-650 nm (peaked at 488 nm), predominantly ^3^P_1_ → ^1^S_0_ transition. The broad, red-shifted profile and multiple shoulders result from site-splitting of Bi^3^⁺ occupying different local environments in the host lattice (Y^3^⁺ gives rise to multiple nonequivalent cation sites — often treated as two main sites, but in practice up to three distinct local environments can be present due to site occupation, defects, or local distortion), plus contributions from CT and defect-related recombination. Intensity of emission spectra from concentration 0.1 mol% to 0.3 mol% increases, after that the intensity of emission spectra decreases regularly for the remaining concentration of Bi ion, as shown in Fig. 8(b). This phenomenon is known as concentration quenching. Phenomenon concentration quenching depends on the critical distance. There are two types of interaction that happen with dopant ions in the host lattice, one is the exchange interaction other is the multipolar interaction. Interaction depends on the R_c_ (critical distance). If the value of R_c_ is less than 5 Å, then energy transfer happens due to exchange interaction, while if R_c_ is greater than 5 Å, then energy transfer happens due to multipolar interaction. R_c_ can be calculated using Bless equation, R_c_ can be calculated by,

1 $$R_{C} = 2\left( {\frac{3V}{{4\prod X_{C} N}}} \right)^{{{\raise0.7ex\hbox{$1$} \!\mathord{\left/ {\vphantom {1 3}}\right.\kern-0pt} \!\lower0.7ex\hbox{$3$}}}}$$

Where V is the volume of the host unit cell, N is the number of cations in the unit cell, and X_c_ is the critical concentration of activator ion^8,32^. For Y₂O₃: Bi, the value of V= 1191.37 Å^3^, N=1, and X_c_ = 0.3. The calculated critical distance is 19.65089503 Å. Energy transfer in the Bi ion is due to multipolar interaction. To evaluate the effect of concentration on emission intensity using Dexter’s theory, using the following equation:

2 $$\log \frac{1}{x} = c - \frac{\theta }{3}\log x$$

Where I is emission intensity and x is concentration of active ions, K, and c is a constant. According to Dexter’s theory, 3 types of multipolar interaction are possible. The type of multipolar interaction depends on the value of θ in equation 2. If the value of θ is 6 for dipole-dipole, 8 for dipole-quadruple, and 10 for quadruple-quadruple. Figure 8(c) shows the Log(I/X) vs Log(X). The value of the slope is found to be -1.29. So the value of θ is 3.87, which is near to 4. So concentration quenching is due exchange interaction^33,34^.

**Fig. 9:**
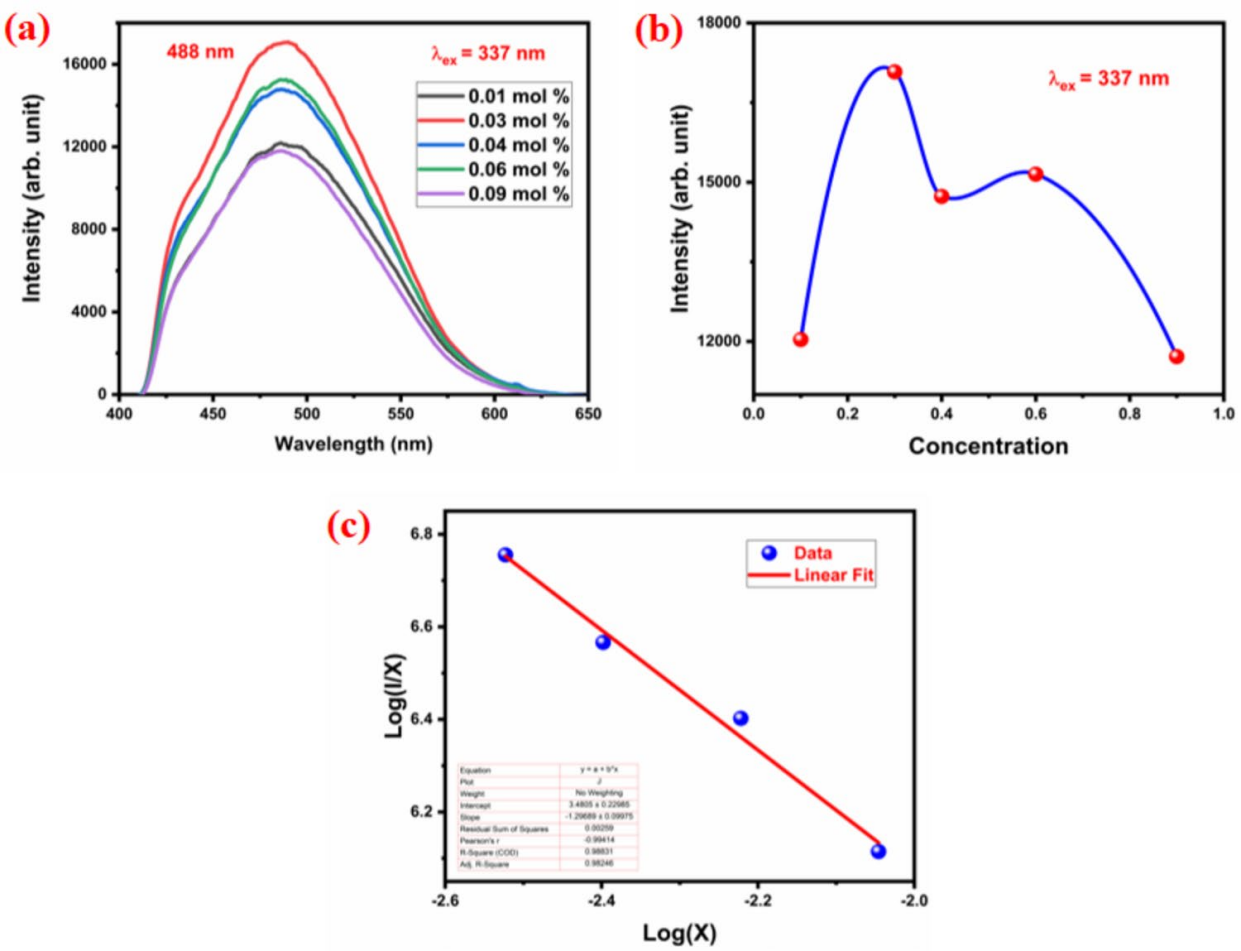
Emission spectra, concentration quenching, and Log(I/X) vs Log(X) of Y₂O₃: Bi at excitation wavelength 337 nm.

Figure 9(a) shows the emission spectra of Y₂O₃: Bi at an excitation wavelength of 337 nm. Emission spectra are recorded in the range from 400 nm to 650 nm. Emission spectra is recorded at different concentrations of Bi ion, such as 0.1, 0.3, 0.4, 0.6, and 0.9 mol%. Emission spectra contain a broad spectral range from 415 nm to 625 nm, centred at 488 nm. The ^1^S_0_→^1^P_1_ transition of Bi^3^⁺ ions—an permitted intra-configurational transition within the 6s^2^ electronic configuration—occurs when Bi^3^-doped Y₂O₃ is stimulated at 337 nm. The higher singlet state (^1^*P*_1_), which is populated by this excitation, rapidly relaxes non-radiatively to the lower triplet level (^3^*P*_1_) via spin–orbit coupling. This triplet level’s radiative decay to the ground state (^3^P_1_→^1^S_0_) produces a wide blue emission band that ranges from 400 to 650 nm, peaking at 488 nm. Concentration of Bi ion at 0.3 mol% has the maximum intensity compared to other concentrations. The effect of concentration is seen in the above emission spectra, before and after the concentration of 0.3 mol% intensity of the emission spectra decreases regularly as shown in Fig. 9(b). For calculating the value of critical distance, the value of V= 1191.37 Å^3^, N=1, and X_c_ = 0.3. The calculated critical distance is 19.65089503 Å. The value of the critical distance is greater than 5 Å, so energy transfer in the Bi ion is due to multipolar interaction. By Dexter’s theory, the value of the slope factor is 1.29, obtained from the graph of Log(I/X) vs Log(X) of Y₂O₃: Bi at excitation wavelength 337 nm, shown in Fig. 9(c). The value of θ is 3.87, which is near to 4. So concentration quenching is due exchange interaction^35^.

When we excite the Y₂O₃: Bi phosphor at 329 nm and 337 nm, the difference between the two emission spectra is the emission intensity and a hump near 430 nm in the emission spectra. The intensity of the emission spectra when it is excited by 329 nm is greater than when it is excited by 337nm. There is a small hump near 430 nm in the emission spectra when it is excited by 337 nm, which is absent in the emission spectra when it is excited by 329 nm.

**Fig. 10:**
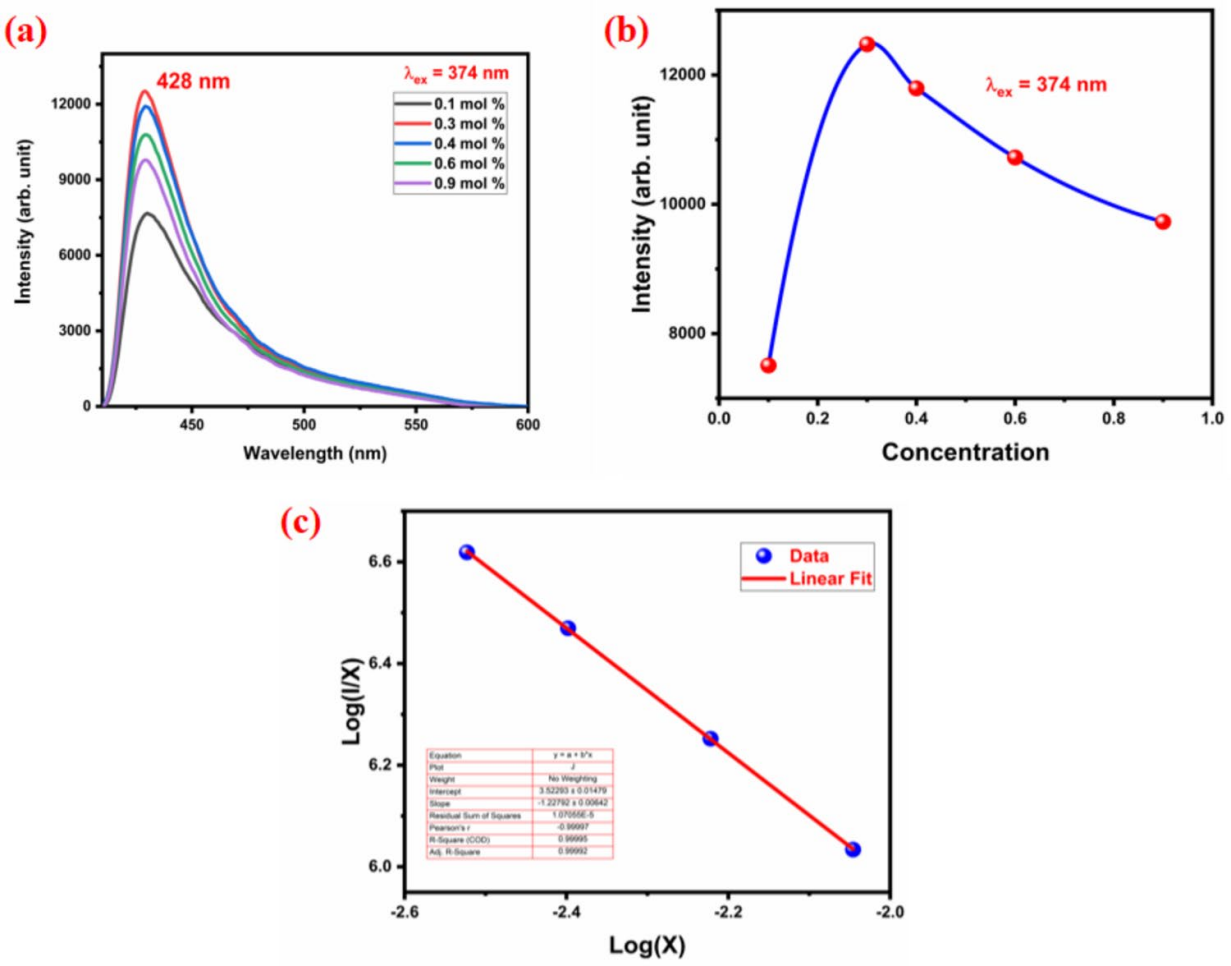
Emission spectra, concentration quenching, and Log(I/X) vs Log(X) of Y₂O₃: Bi at excitation wavelength 428 nm.

Emission spectra of Y₂O₃: Bi at excitation wavelength 374 nm are shown in Figure 10(a). Emission spectra are recorded from 400 nm to 600 nm for the different concentrations of Bi, such as 0.1, 0.3, 0.4, 0.6, and 0.9 mol%. Emission spectra contain a broad spectrum mainly located in the intense blue region. Emission spectra of Y₂O₃: Bi are from 400 nm to 600 nm, centred at 428 nm. Bi^3^⁺ ions in Y₂O₃ are excited at 374 nm and go through the ^1^S_0_ →^3^P_1_ transition, which is spin-forbidden but becomes partially allowed because of Bi^3^’s⁺ strong spin–orbit interaction. The radiative degradation from the ^3^*P*_1_ that follows reaching the ^1^*S*_0_ state results in blue emission with a 428 nm centre. Bi^3^⁺ replaces Y^3+^ at two nonequivalent locations in the cubic bixbyite Y₂O₃ lattice, non-centrosymmetric C₂ and centrosymmetric C₃ᵢ. This results in minor changes in the local crystal field. Due to its greater dipole transition probability, Bi^3^⁺ ions at the C₂ site are primarily responsible for the increased emission at 428 nm. Local distortions and contributions from the C₃ᵢ site result in some broadening and asymmetry in the emission band. Emission intensity is maximum at 0.3 mol% after and before that, the emission intensity decreases as shown in Fig. 10(b). This is due to concentration quenching. Concentration quenching depends on the critical distance. For calculating the the value of critical distance, the value of V= 1191.37 Å^3^, N=1, and X_c_ = 0.3. The calculated critical distance is 19.65089503 Å. The value of the critical distance is greater than 5 Å, so energy transfer in the Bi ion is due to multipolar interaction. By Dexter’s theory, the value of the slope factor is 1.22 from the graph of Log(I/X) vs Log(X) of Y₂O₃: Bi at excitation wavelength 428 nm, as shown in Fig. 10(c). The value of θ is 3.66, which is near to 4. So concentration quenching is due exchange interaction.

Comparing 3 emission spectra, the Emission spectra at excitation wavelength 329 nm have both blue and green components in the emission spectra. Emission spectra at excitation wavelength 337 nm have both blue and green components in the emission spectra, with a slightly more blue component that is greater than 329 nm. Emission spectra at 374 nm have majorly blue components in emission spectra with negligible green components.

**Fig. 11:**
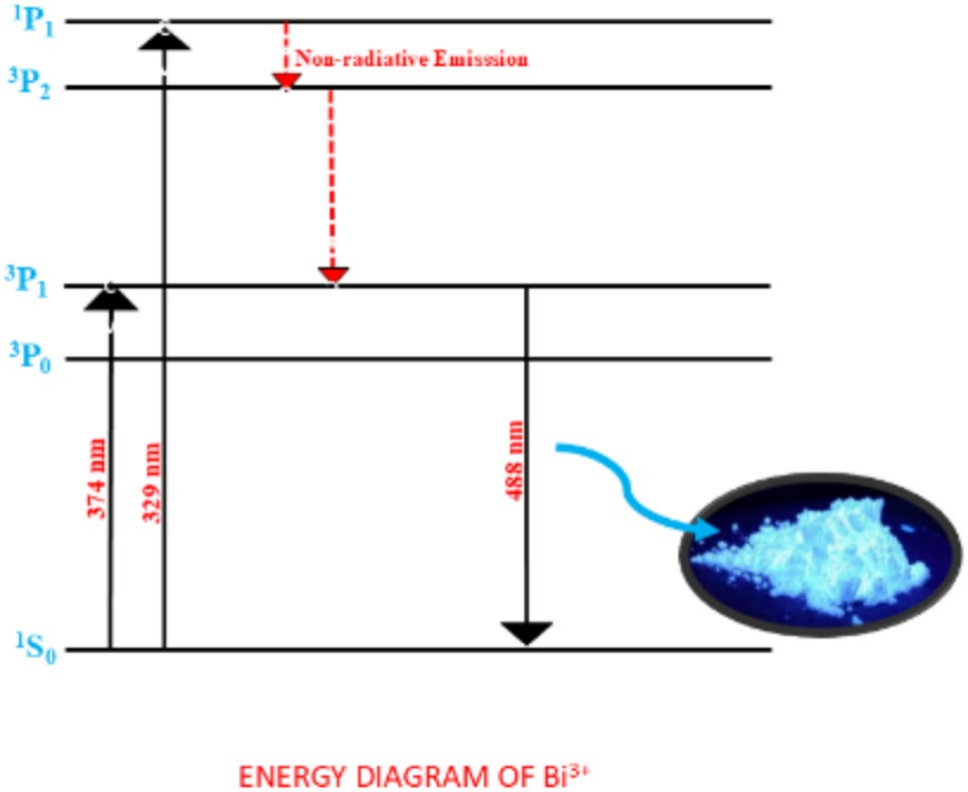
Energy level diagram of Y₂O₃: Bi.

Figure 11 shows the energy level diagram of Bi ion. Above transition are responsible for formation of peak . Board nature due the splitting of energy level . With splitting the oxygen vacancy contribute in board spectra. At excitation 374 nm , emission spectra is less board as compare at 337 nm and 327 nm. This may be happen due to the less contribution in emission spectra by oxygen vacancy at 374 nm because with changing excitation wavelength only emission from oxygen vacancy is changes other all parameter such as cite symmetry, transition , and surrounding environment.

#### Y₂O₃: Bi/Li

**Fig. 12:**
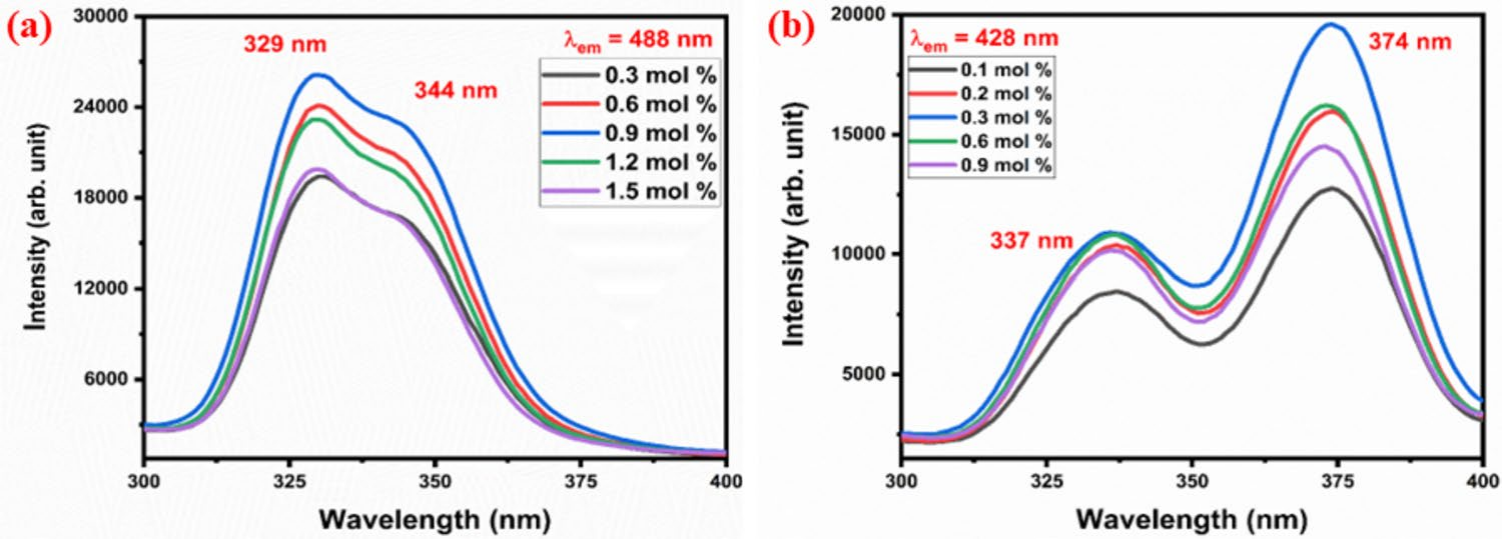
Excitation spectra of Y₂O₃: Bi/Li at emission wavelengths 428 nm and 488 nm.

Figure 12(a) and 12(b) show the excitation spectra of Y₂O₃: Bi/Li at emission wavelengths 428 nm and 488 nm, respectively. The concentration of Bi ion is kept constant at 0.3 mol% due to its highest emission intensity, and we vary the concentration of Li ion from 0.3 mol% to 1.5 mol%. Excitation spectra at emission wavelength 488 nm are recorded from 300 nm to 400 nm. The spectra are wide and have two humps between 300 and 375 nm. Humped at 329 nm has a higher intensity than humped at 344 nm. This board spectra due to the spin-allowed ^1^S₀ → ^1^P₁ transition of Bi^3^⁺ ions occupying the two nonequivalent cation sites (C₂ and C₃ᵢ) in the cubic lattice is responsible for the excitation humps at ~329 nm and ~344 nm. Strong electron-phonon coupling, spectral overlap with the O^2^⁻→Bi^3^⁺ charge-transfer band, and the presence of several low-symmetry sites are responsible for the substantial spectral breadth, with the dual humps are caused by the crystal-field splitting of the ^1^P₁ state at these different sites. By serving as a charge compensator and encouraging the more effective incorporation of Bi^3^⁺ into the Y₂O₃ lattice and passivating non-radiative defect centres, the addition of Li⁺ co-dopant mainly increases the intensity of these excitation bands. Without significantly altering the transition energies, this raises the quantity of optically active Bi^3^⁺ ions. Excitation spectra at emission wavelength 488 nm are recorded from 300 nm to 400 nm. Excitation spectra contain two peaks at 337 nm and 374 nm. Both peak has a broad nature. The excitation spectrum of Bi^3^⁺-doped Y₂O₃ at 428 nm emission shows two peaks at 337 nm and 374 nm, which correspond to the ^1^S_0_ →^1^P_1_ (spin-allowed) and ^1^S_0_→^3^P_1_ (spin-forbidden)^36^

**Fig. 13:**
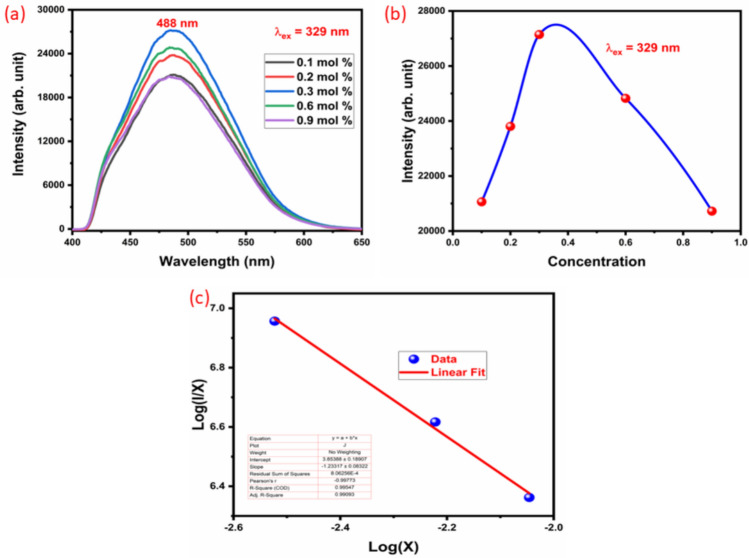
Emission spectra, concentration quenching, and Log(I/X) vs Log(X) of Y₂O₃: Bi/Li at excitation wavelength 329 nm.

Figure 13 (a) shows the emission spectra of Y₂O₃: Bi/Li at an excitation wavelength at 329 nm recorded from 400 nm to 650 nm. Emission spectra are recorded at different concentrations of Li ions, such as 0.3 mol%, 0.6 mol%, 0.9 mol%, 1.2 mol%, and 1.5 mol%. Emission spectra contain a broad spectrum from near 415 nm to 650 nm, centred at 488 nm. The primary source of this emission is the Bi^3^⁺ ions’ ^3^P_1_→^1^S_0_ transition, with additional contributions from charge-transfer and defect-related recombination. Site-splitting and local crystal-field variations are caused by Bi^3^⁺ occupying several nonequivalent Y^3^⁺ sites (two major and up to three local environments) in the cubic Y₂O₃ lattice, which accounts for the broad profile. Emission spectra contain both blue and green component in the emission spectra. There is no such extra peak or shifting of peak due to Li ions being absorbed in the above emission spectra. It only increases the PL emission intensity of the prepared phosphor.^37^ In the prepared phosphor, the Li ion serves as a charge compensator. As seen in Fig. 13 (b), the emission spectrum intensity reaches its maximum at 0.3 mol%, after which the PL intensity steadily declines. This phenomenon is known as concentration quenching, which depends on the critical distance. In this case, to find the critical distance, the value of V= 1198.327 Å^3^, N=1, and X_c_ = 0.9. The calculated critical distance is 13.65163954 Å. The value of critical distance is greater than 5 Å, so energy transfer in the Bi ion is due to multipolar interaction. By Dexter’s theory, the value of the slope factor is 1.23 from the graph in Fig. 13 (c). The value of θ is 3.69, which is near to 4. So concentration quenching is due exchange interaction.

**Fig. 14:**
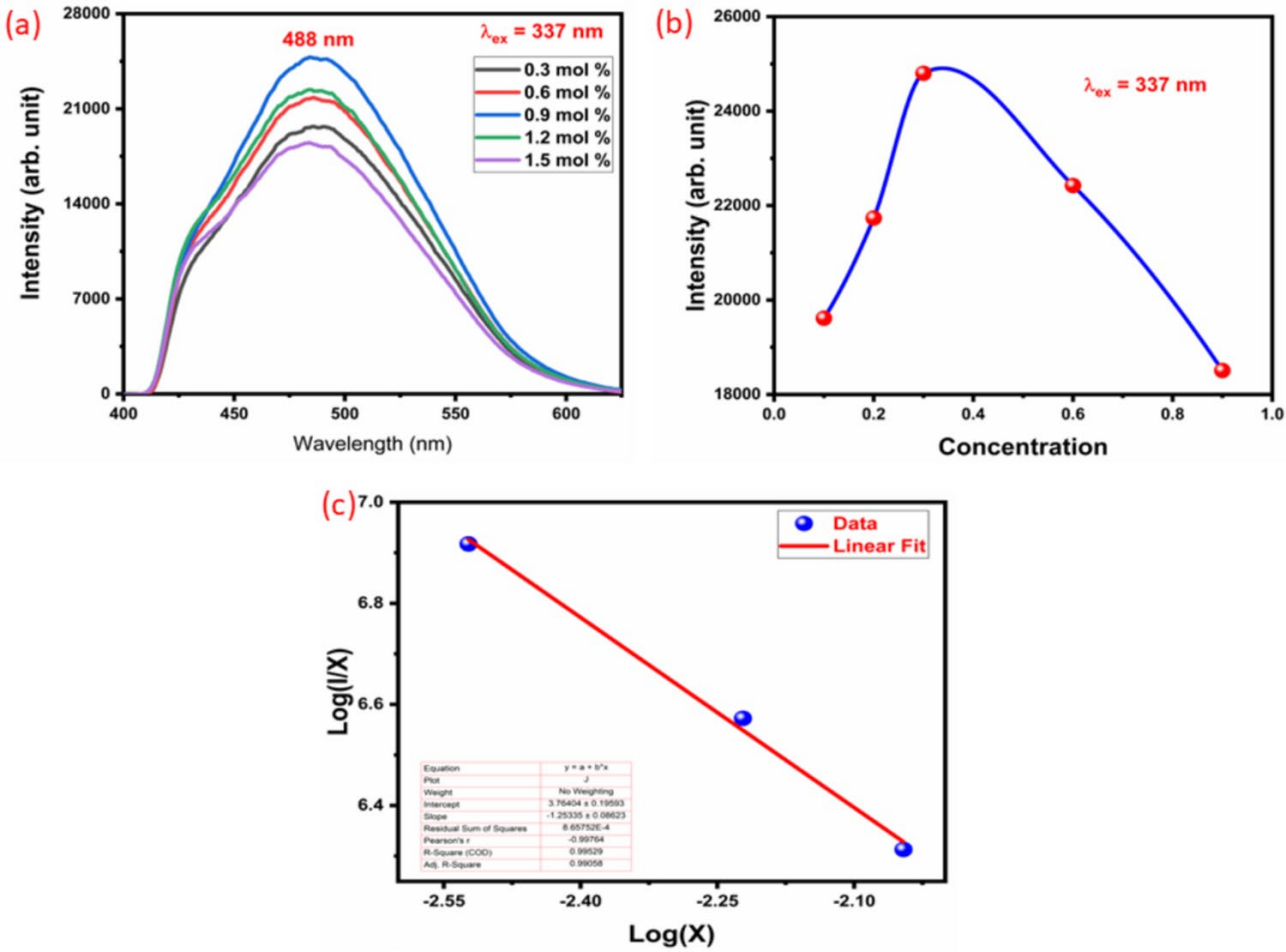
Emission spectra, concentration quenching, and Log(I/X) vs Log(X) of Y₂O₃: Bi/Li at excitation wavelength 337 nm.

Emission spectra of Y₂O₃: Bi/Li are obtained at an excitation wavelength of 337 nm, covering the range from 400 nm to 650 nm, as illustrated in Fig. 14 (a). Emission spectra recorded for different concentrations of Li ions from 0.3 mol% to 1.5 mol% with a constant interval of 0.3 mol%. Like Y₂O₃: Bi, emission spectra at excitation wavelength 337 nm have the same behaviour as the previous. Emission spectra range from 415 nm to 650 nm, centred at 488 nm with humped near 433 nm. Bi^3^⁺-doped Y₂O₃ exhibits a comparable wide emission (400–650 nm, centred at 488 nm) under 337 nm excitation, which corresponds to the ^3^P_1_→^1^S_0_ transition^38^. Because Bi^3^⁺ ions occupy locations with the same symmetry but undergo modest fluctuations in local crystal-field environments within the Y₂O₃ lattice, a little hump forms near 430 nm. Variation of PL emission intensity as a function of concentration of Li ion is shown in Fig. 14 (b).To find the critical distance , the value of V= 1198.327 Å^3^, N=1, and X_c_ = 0.9. The calculated critical distance is 13.65163954 Å. The value of critical distance is greater than 5 Å so energy transfer in Bi ion is due to multipolar interaction. Graph of Log(I/X) vs Log(X) of Y₂O₃: Bi/Li is shown in Fig. 14(c). By Dexter’s theory, the value of slope factor is 1.25. The value of θ is 3.75, which is near to 4. So concentration quenching is due exchange interaction.

**Fig. 15:**
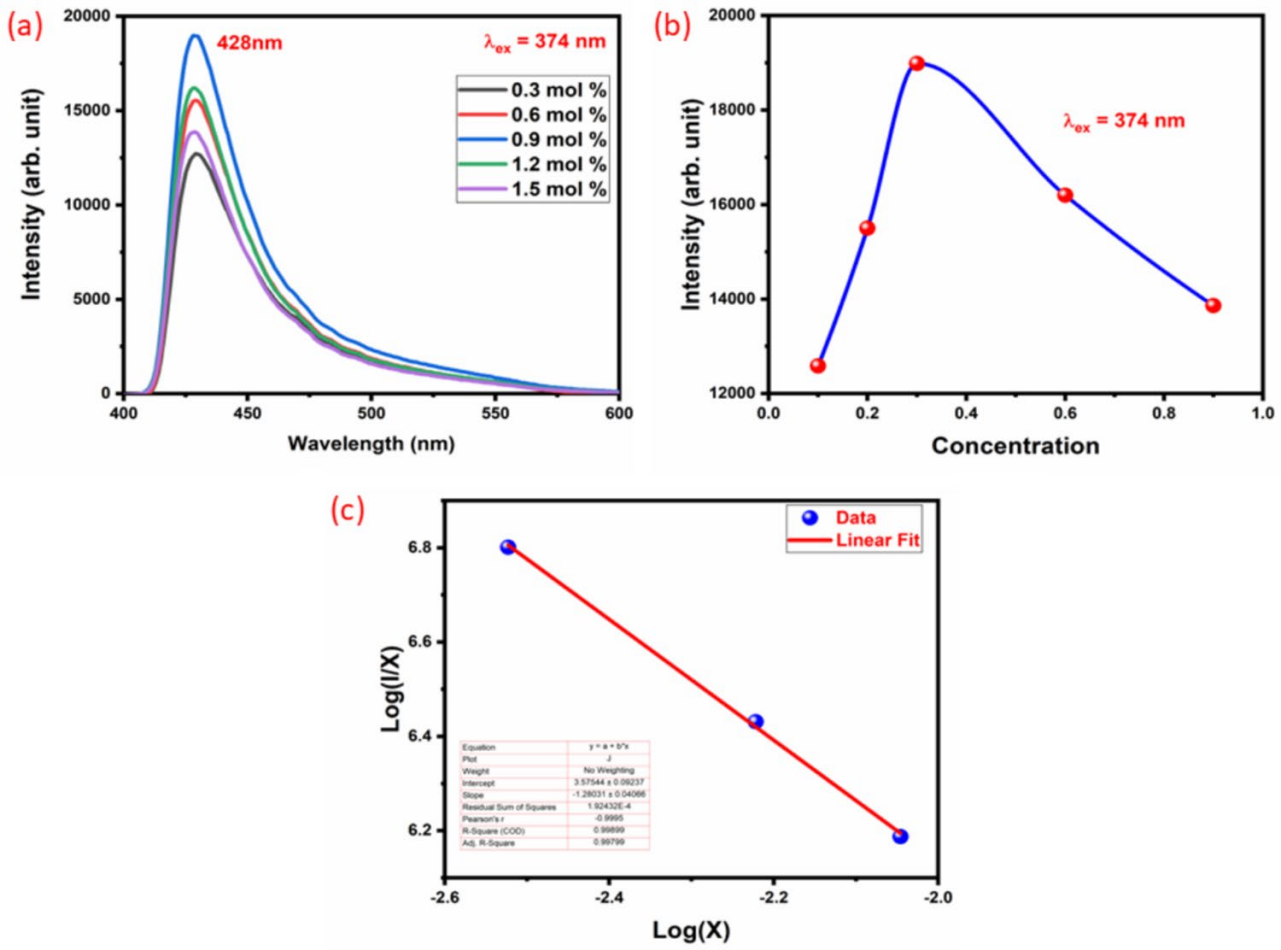
Emission spectra, concentration quenching, and Log(I/X) vs Log(X) of Y₂O₃: Bi/Li at excitation wavelength 374 nm.

Figure 15 (a) shows the emission spectra of Y₂O₃: Bi/Li at excitation wavelength 374 nm recorded from 400 nm to 600 nm. Emission peak in the same board nature ranges from 410 nm to 600 nm, centred at 428 nm, same as Y₂O₃ : Bi. This confirms the emission spectra are not shifted or add any other peaks due to the addition of the Li ion. Doping of Li ion increases the PL emission intensity. spin-forbidden but prtially permitted by strong spin-orbit coupling); the associated ^3^P_1_→^1^S_0_ radiative decay is the observed 428 nm emission. Small shifts or shoulders in the band are induced by site-splitting, local crystal-field variations (phonon sidebands), and potential neighboring defect/CT contributions. The emitting Bi^3^⁺ ions are located on the Y^3^⁺ sites of the cubic bixbyite lattice (mostly C₂ and C₃ᵢ symmetries). As we see the emission intensity versus concentration of Li ion, concentration 0.9 mol% as the maximum PL intensity before and after that, PL intensity decreases (Fig. 15 (b)). This phenomenon is due to concentration quenching. To find critical distance To find the critical distance, the value of V= 1198.327 Å^3^, N=1, and X_c_ = 0.9. The calculated critical distance is 13.65163954 Å. The value of critical distance is greater than 5 Å so energy transfer in Bi ion is due to multipolar interaction. Graph of Log(I/X) vs Log(X) of Y₂O₃: Bi/Cs is shown in Fig. 15(c). By Dexter’s theory, the value of slope factor is 1.26. The value of θ is 3.78, which is near to 4. So concentration quenching is due exchange interaction.

#### Y₂O₃: Bi/K

**Figure. 16:**
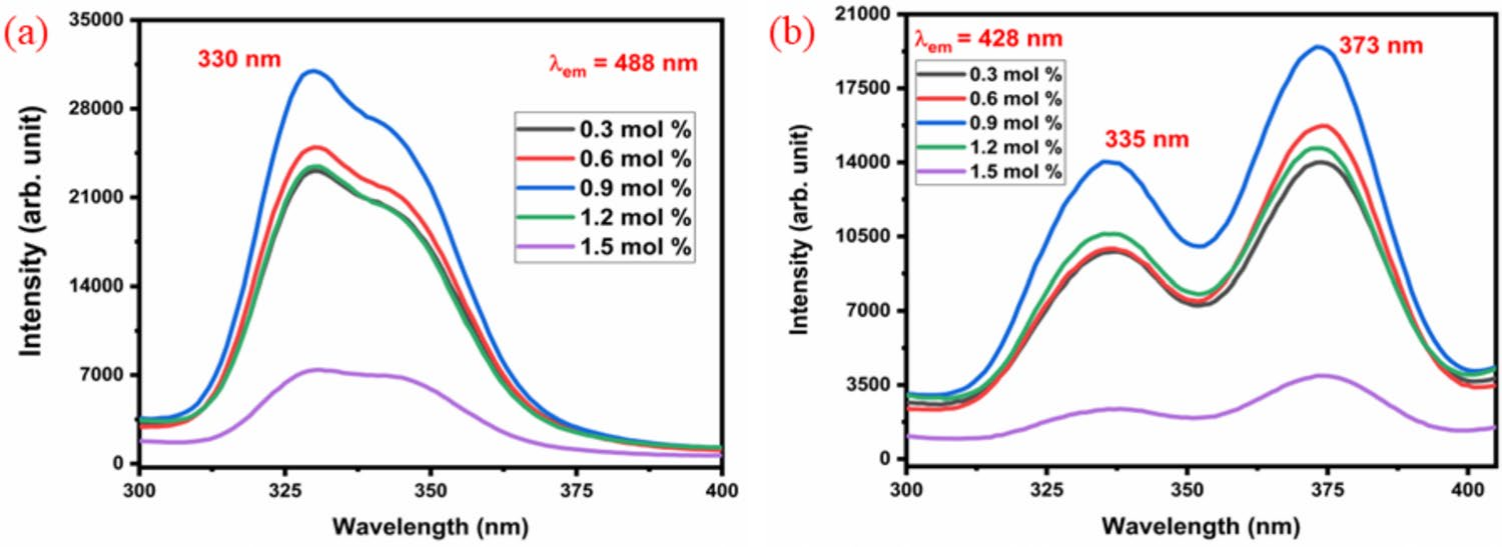
Excitation spectra of Y₂O₃: Bi/Na at emission wavelengths 428 nm and 488 nm.

Figure 16(a), (b) displays the excitation spectra of Y₂O₃: Bi/Na for emission wavelengths 428 nm and 488 nm. Maintaining the Bi ion concentration at 0.3 mol%, we continue to alter the concentration of K from 0.3 to 1.5 mol%. Excitation spectra at emission wavelength 488 nm contain one broad spectrum from 315 nm to 375 nm with humped at 330 nm and 344 nm. This board peak is due to the ^1^S₀ → ^1^P₁ transition overlapping with the charge-transfer band. Excitation spectra at emission spectra 428 nm, contain 2 broad peaks at 335 nm and 373 nm due to the ^1^S_0_ →^1^P_1_ (spin-allowed) and ^1^S_0_→^3^P_1_ (spin-forbidden)

**Fig. 17:**
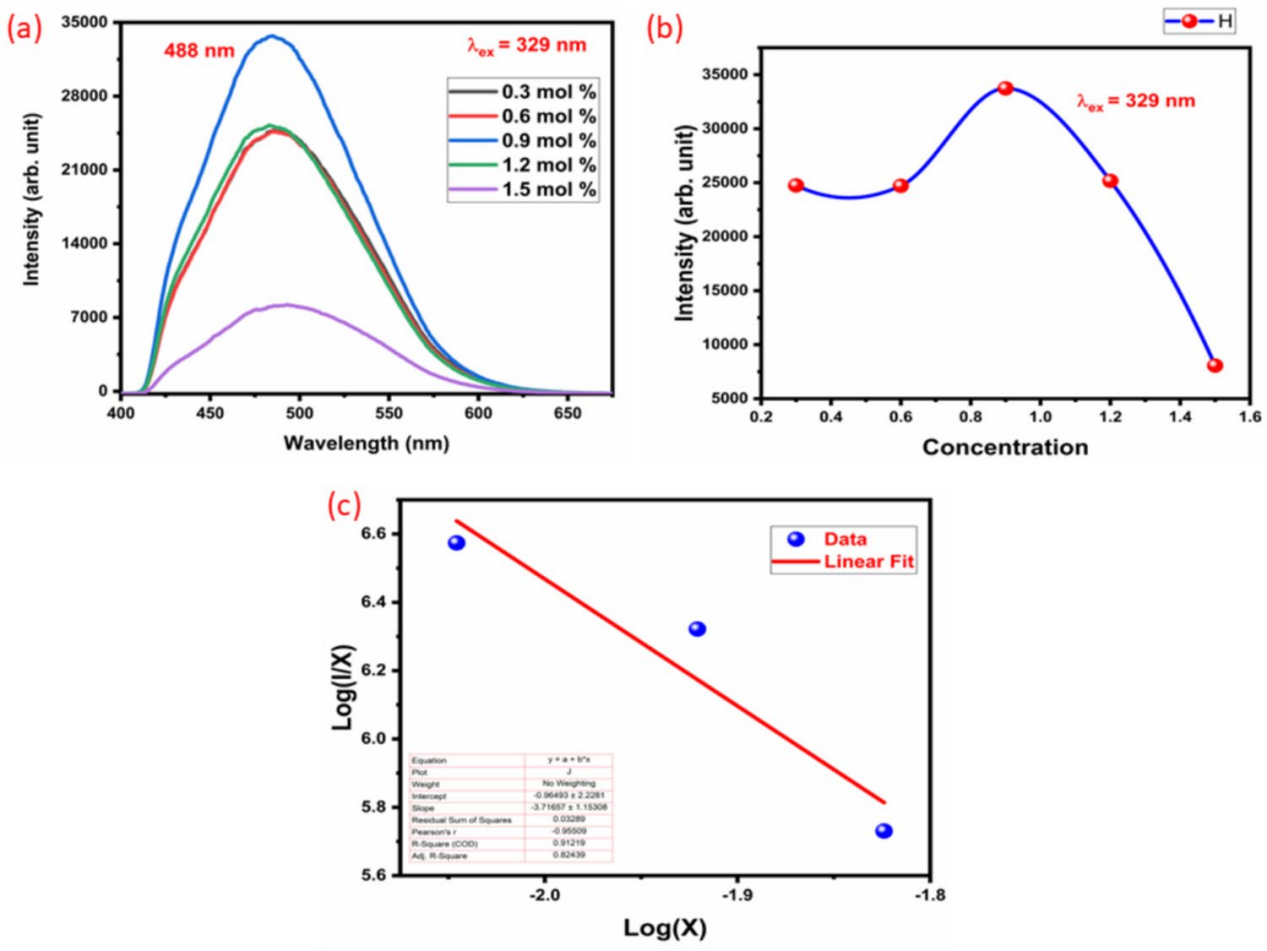
Emission spectra, concentration quenching, and Log(I/X) vs Log(X) of Y₂O₃: Bi/K at excitation wavelength 329 nm.

Figure 17(a) shows the emission spectra of Y₂O₃: Bi/K at excitation wavelength 329 nm recorded from 400 nm to 650 nm. Emission spectra are recorded for 5 different concentrations of Cs ion from 0.3 mol% to 1.5 mol% with a constant variation of 0.3 mol% by keeping the concentration of Bi ion at 0.3 mol%. Emission spectra of the same nature as Y₂O₃: Bi that confirm the absence of the effect of doping of K in terms of shifting and adding new peaks. It only affects the change in the intensity of the prepared phosphor. Emission spectra contain a broad range from 415 nm to 650 nm, centred at 488 nm. The primary source of this emission is the Bi^3^⁺ ions’ ^3^P_1_→^1^S_0_ transition, with additional contributions from charge-transfer and defect-related recombination. Site-splitting and local crystal-field variations are caused by Bi^3^⁺ occupying several nonequivalent Y^3^⁺ sites (two major and up to three local environments) in the cubic Y₂O₃ lattice, which accounts for the broad profile. When the concentration of K ion is raised from 0.3 mol% to 0.9 mol%, the intensity of PL emission consistently rises, reaching its peak at 0.9 mol%. After 0.9 mol%, PL emission intensity decreases regularly, as shown in Fig. 17(b). To find the critical distance, V= 1187.344 Å^3^, N=1, and X_c_ = 0.9. The calculated critical distance is 13.60980444 Å. The value of critical distance is greater than 5 Å so energy transfer in Bi ion is due to multipolar interaction. By Dexter’s theory, the value of slope factor is 3.7 from the graph in Fig. 17(c). The value of θ is 11, which is near to 14.8. So concentration quenching is due quadruple- quadruple interaction.

**Fig. 18:**
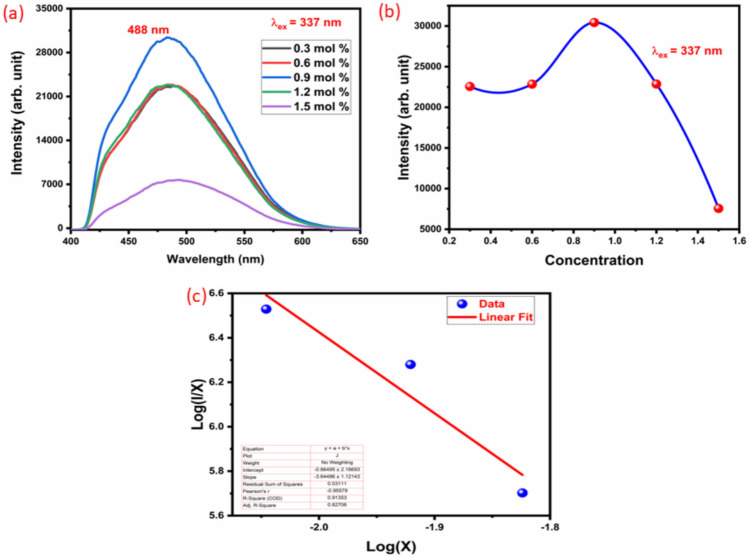
Emission spectra, concentration quenching, and Log(I/X) vs Log(X) of Y₂O₃ : Bi/K at excitation wavelength 337 nm.

The emission spectra of Y₂O₃: Bi/K have been captured using an excitation wavelength of 337 nm, as depicted in Fig. 18(a). The emission spectra display a wide peak centered around 488 nm, along with a slight hump near 430 nm. Board spectra correspond to the ^3^P_1_→^1^S_0_ transition. Bi^3^⁺ ions occupying positions of the same symmetry but experiencing modest differences in local crystal-field environments within the Y₂O₃ lattice cause a slight hump to form about 430 nm. Concentration quenching happens at a concentration of 0.9 mol% from Fig. 18(b). For finding critical distance, the value of V= 13.60980444 Å^3^, N=1, and X_c_ = 0.9. The calculated critical distance is 19.65089503 Å. The value of critical distance is greater than 5 Å from the graph in Fig. 18(c). So energy transfer in Bi ion is due to multipolar interaction. By Dexter’s theory, the value of slope factor is 3.64. The value of θ is 10.92, which is near to 10. So concentration quenching is due quadruple- quadruple.

**Fig. 19:**
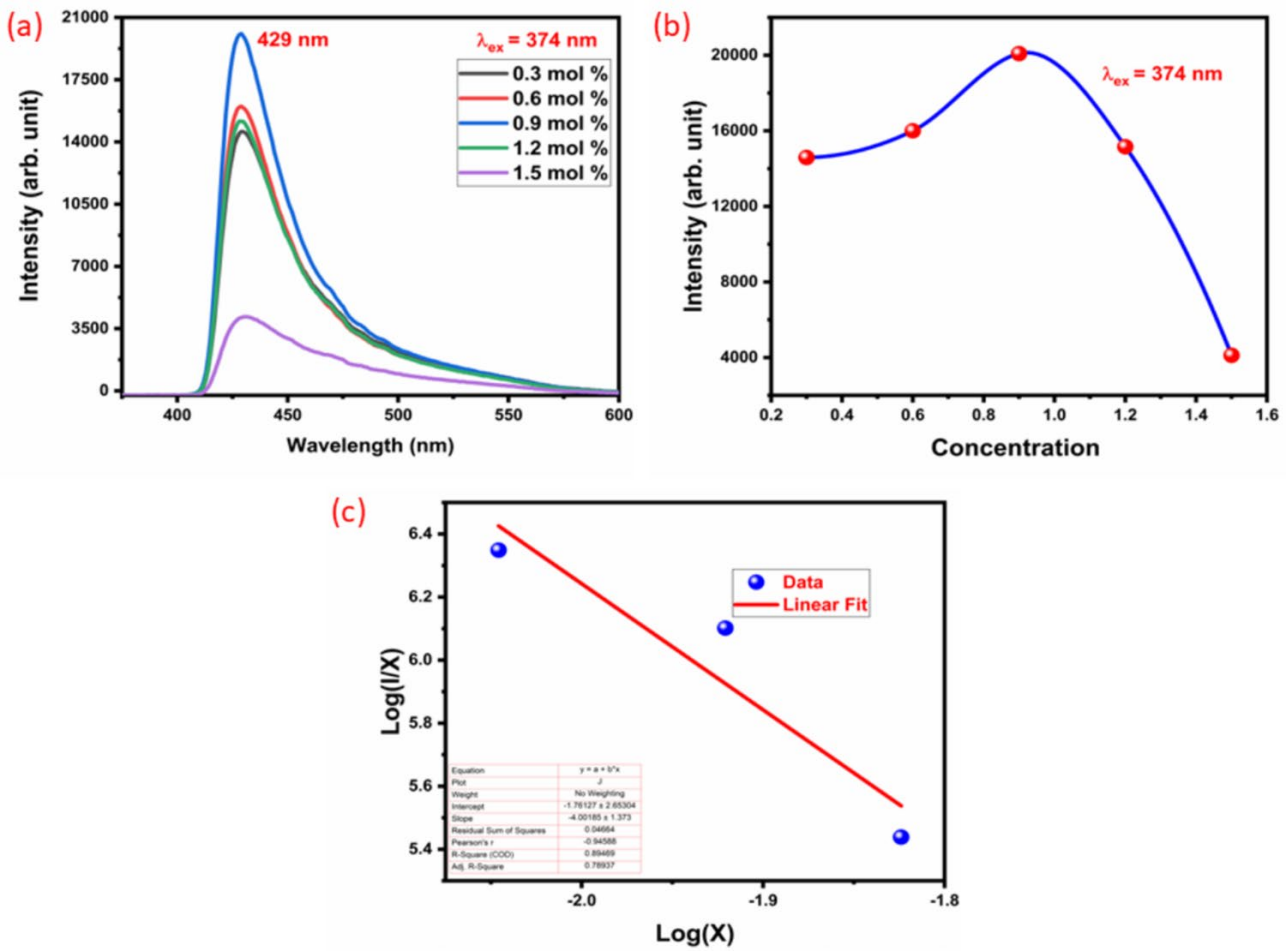
Emission spectra, concentration quenching, and Log(I/X) vs Log(X) of Y₂O₃ : Bi/K at excitation wavelength 374 nm.

Emission spectra at excitation wavelength 429 nm are shown in Fig. 19(a). emission spectra contain one intense blue color emitting a broad peak centred at 429 nm. The measured 428 nm emission is the corresponding ^3^P_1_→^1^S_0_ radiative decay; spin-forbidden but partially allowed by strong spin–orbit coupling. The Y^3^⁺ sites of the cubic bixbyite lattice (mostly C₂ and C₃ᵢ symmetries) are where the emitting Bi^3^⁺ ions are located. Site-splitting, local crystal-field changes (phonon sidebands), and potential neighboring defect/CT contributions are responsible for tiny shifts or shoulders in the band. Doping of K only affects the intensity of the emission peak; parameter like shifting or any other peaks is absent. Concentration quenching happens at a concentration of 0.9 mol% of the K ion, as shown in Fig. 19(b). Figure19 (c) shows the Log(I/X) vs Log(X) of Y₂O₃: Bi/K. To find the critical distance , the value of V= 13.60980444 Å^3^, N=1, and X_c_ = 0.9. The calculated critical distance is 19.65089503 Å. The value of critical distance is greater than 5 Å so energy transfer in Bi ion is due to multipolar interaction. By Dexter’s theory, the value of slope factor is 4.0. The value of θ is 12, which is near to 10. So concentration quenching is due quadruple- quadruple.

#### Y₂O₃: Bi/Na

**Fig. 20:**
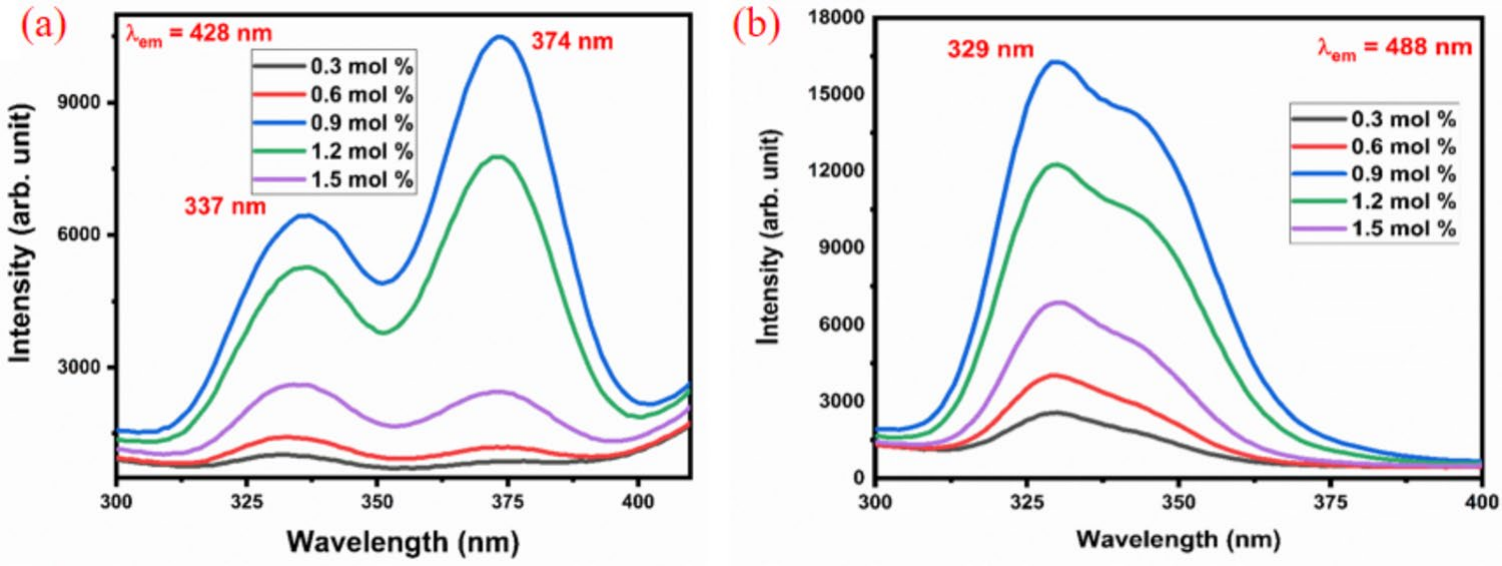
Excitation spectra of Y₂O₃: Bi/Na at emission wavelengths 428 nm and 488 nm.

The excitation spectra of Y₂O₃: Bi/Na at emission wavelengths of 428 nm and 488 nm are displayed in Fig. 20(a) and (b). By keeping the concentration of Bi ion at 0.3 mol% constant, we keep varying the concentration of Na ion from 0.3 mol% to 1.5 mol%. Excitation spectra at emission wavelength 488 nm have the same behaviour as Y₂O₃: Bi with two humped at 329 nm and 344 nm. This peak is due to the ^1^S₀ → ^1^P₁ transition, which overlaps with the charge transfer band. The excitation spectrum of Bi^3^⁺-doped Y₂O₃ at 428 nm emission shows two peaks at 337 nm and 374 nm, which correspond to the ^1^S_0_ →^1^P_1_ (spin-allowed) and ^1^S_0_→^3^P_1_ (spin-forbidden)

**Fig. 21:**
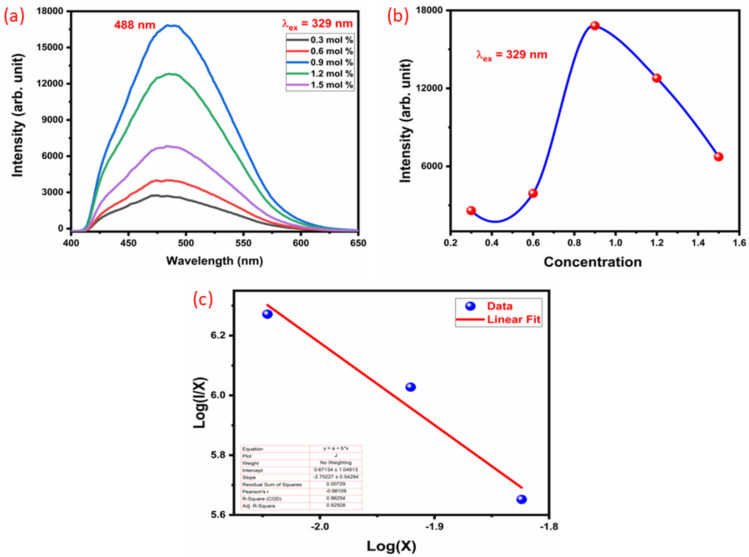
Emission spectra, concentration quenching, and Log(I/X) vs Log(X) of Y₂O₃: Bi/Na at excitation wavelength 329 nm.

Figure 21 (a) displays the emission spectra of Y₂O₃: Bi/Na at excitation wavelength 329. At various Na concentrations ranging from 0.3 mol% to 1.5 mol%, emission spectra are captured. The emission spectrum is centred at 488 nm and spans a wide range from 415 to 650 nm. While the ^3^P_1_→^1^S_0_ transition of Bi^3^⁺ ions is the main source, charge-transfer and defect-related recombination also contribute to this emission. In the cubic Y₂O₃ lattice, Bi^3^⁺ occupies numerous nonequivalent Y^3^⁺ sites (two major and up to three local environments), resulting in site-splitting and local crystal-field fluctuations, which are responsible for the broad profile. Adding Na only affects the intensity of emission PL intensity. variation of intensity of Y₂O₃: Bi/Na is shown in Fig. 21 (b). At 0.9 mol% has maximum intensity before and afterb that PL emission intensity is decreases regularly. This is due to concentration quenching. To find the critical distance, the value of V= 1188.663 Å^3^, N=1, and X_c_ = 0.9. The calculated critical distance is 13.61484221 Å. The value of critical distance is greater than 5 Å so energy transfer in Bi ion is due to multipolar interaction. Figure 21(c) shown the graph of Log(I/X) vs Log(X). By Dexter’s theory, the value of slope factor is 2.75. The value of θ is 8.25, which is near to 8. So concentration quenching is due dipole - quadruple.

**Fig. 22:**
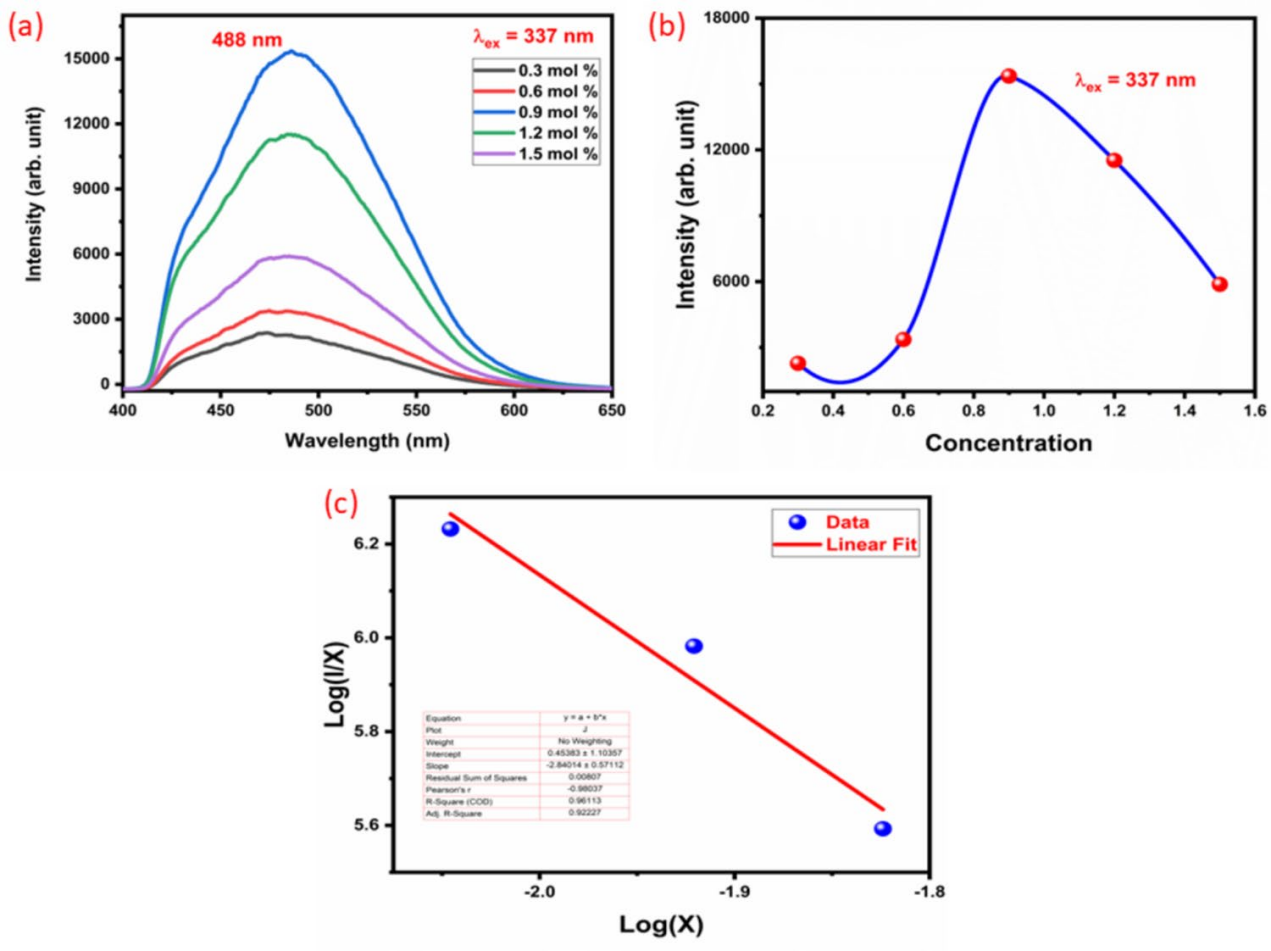
Emission spectra, concentration quenching, and Log(I/X) vs Log(X) of Y₂O₃: Bi/Na at excitation wavelength 337 nm.

Above same phosphor is excited by an excitation wavelength of 337 nm resultant emission spectra are shown in Fig. 22(a). Emission spectra contain a broad spectrum with humped at 430 nm and centred at 488 nm. The ^3^P_1_→^1^S_0_ transition is represented by a similar wide emission (400–650 nm, centred at 488 nm) in Bi^3^⁺-doped Y₂O₃ under 337 nm illumination. Bi^3^⁺ ions occupying positions of the same symmetry but undergoing small changes in local crystal-field environments within the Y₂O₃ lattice cause a slight hump to develop about 430 nm. The concentration of 0.9 mol% has the maximum intensity compared to other concentrations. Figure 22(b) shows the concentration quenching of Y₂O₃: Bi/Na. Adding Na affects the emission intensity. Figure 22(c) shows the graph of Log(I/X) vs Log(X). This is due to concentration quenching. To find the critical distance, the value of V= 13.61484221 Å^3^, N=1, and X_c_ = 0.9. The calculated critical distance is 19.65089503 Å. The value of critical distance is greater than 5 Å, so energy transfer in the Bi ion is due to multipolar interaction. By Dexter’s theory, the value of the slope factor is 2.6. The value of θ is 8.25, which is close to 7.8. So concentration quenching is due dipole – quadruple.

**Fig. 23:**
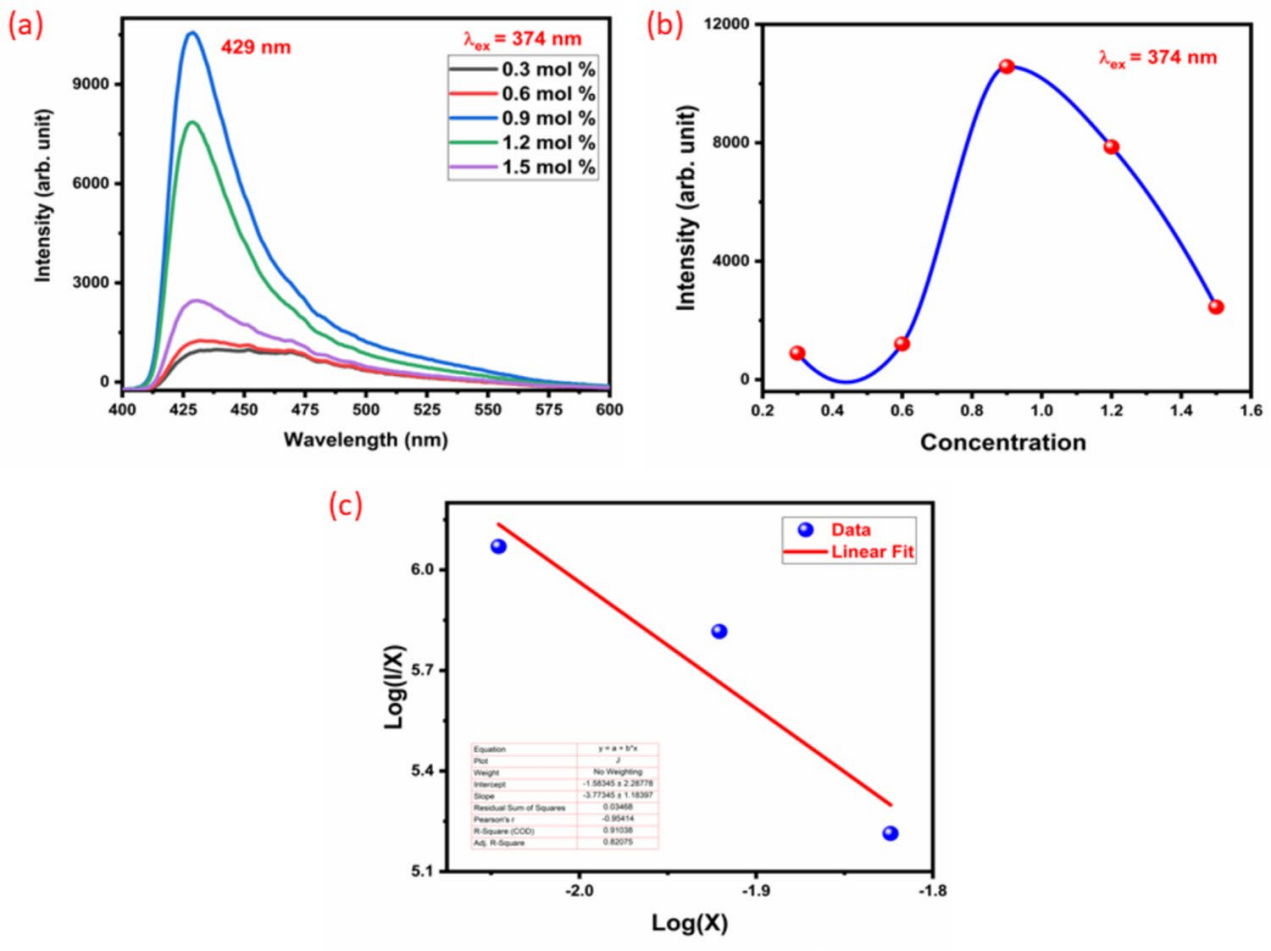
Emission spectra, concentration quenching, and Log(I/X) vs Log(X) of Y₂O₃: Bi/Na at excitation wavelength 374 nm.

Above same phosphor is excited by excitation wavelength 337 nm resultant emission spectra is shown in Fig. 23(a). Emission spectra contain one board spectra with humped at 430 nm and centered at 488 nm. The 3*P*1→1*S*0 transition is represented by a similar wide emission (400–650 nm, centered at 488 nm) in Bi^3^⁺-doped Y₂O₃ under 337 nm illumination. Bi^3^⁺ ions occupying positions of the same symmetry but undergoing small changes in local crystal-field environments within the Y₂O₃ lattice cause a slight hump to develop about 430 nm. Concnetration of 0.9 mol% has maximum intensity as compare to other concentration. Figure 23(b) shown the concentration quenching of Y₂O₃: Bi/Na. Adding Na effect the emission intensity. Figure 23(c) is shown the graph of Log(I/X) vs Log(X). This is due to concentration quenching. To find the critical distance, the value of V= 13.61484221 Å^3^, N=1, and X_c_ = 0.9. The calculated critical distance is 19.65089503 Å. The value of critical distance is greater than 5 Å so energy transfer in Bi ion is due to multipolar interaction. By Dexter’s theory, the value of slope factor is 2.6. The value of θ is 8.25, which is near to 7.8. So concentration quenching is due dipole – quadruple.

#### Y₂O₃: Bi/Cs

**Fig. 24:**
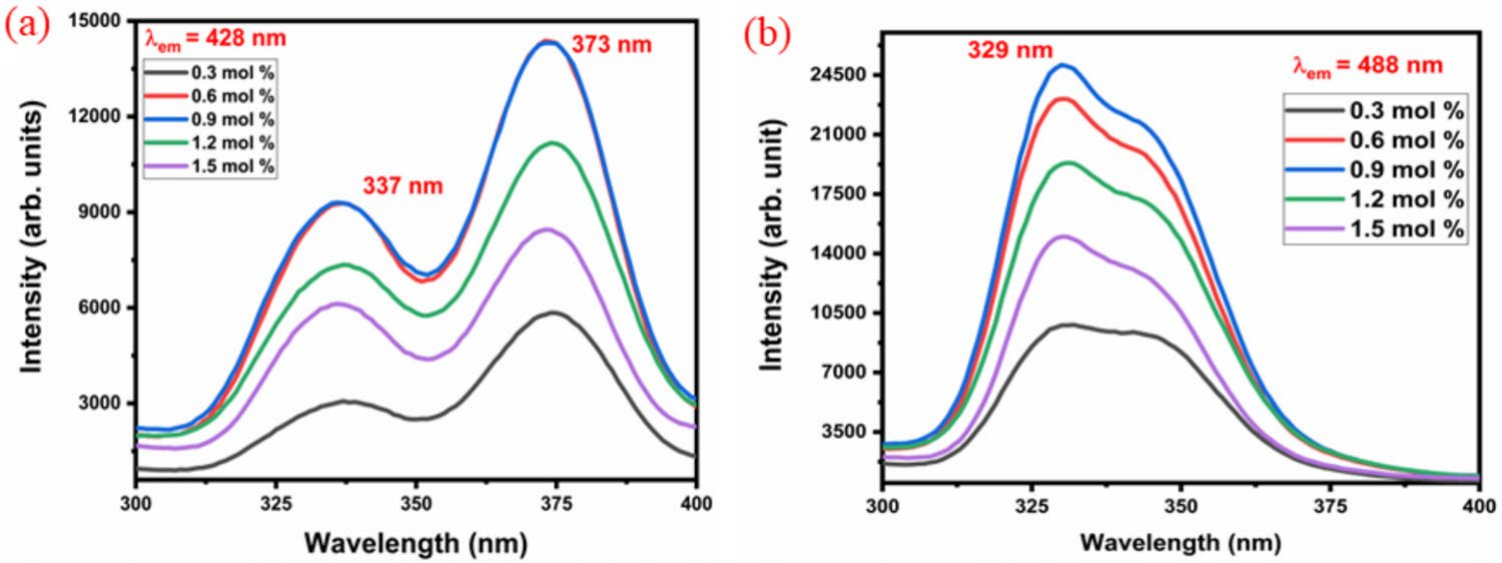
Excitation spectra of Y₂O₃: Bi/Cs at emission wavelengths 428 nm and 488 nm.

Excitation spectra of Y₂O₃: Bi/Cs at emission wavelengths 428 nm and 488 nm are shown in Fig. 24(a) and (b), respectively. Excitation spectra at emission wavelength 428 nm have the same behaviour as before. Y₂O₃: Bi has maximum intensity at 0.3 mol% of the Bi ion, so we keep the concentration of Bi constant. We varied the concentration of Cs ions from 0.3 mol% to 1.5 mol% with a constant interval of 0.3 mol%. Excitation spectra at emission wavelength 428 contain 2 broad peaks centred at 337 nm and 373 nm. This board peak corresponds to the ^1^S_0_ →^1^P_1_ (spin-allowed) and ^1^S_0_→^3^P_1_ (spin-forbidden)_._ Excitation spectra at emission wavelength 488 nm have a broad spectrum from 315 nm to 375 nm with 2 humped at 329 nm and 344 nm, with the S₀ → ^1^P₁ transition overlapping with the charge transfer band.

**Fig. 25:**
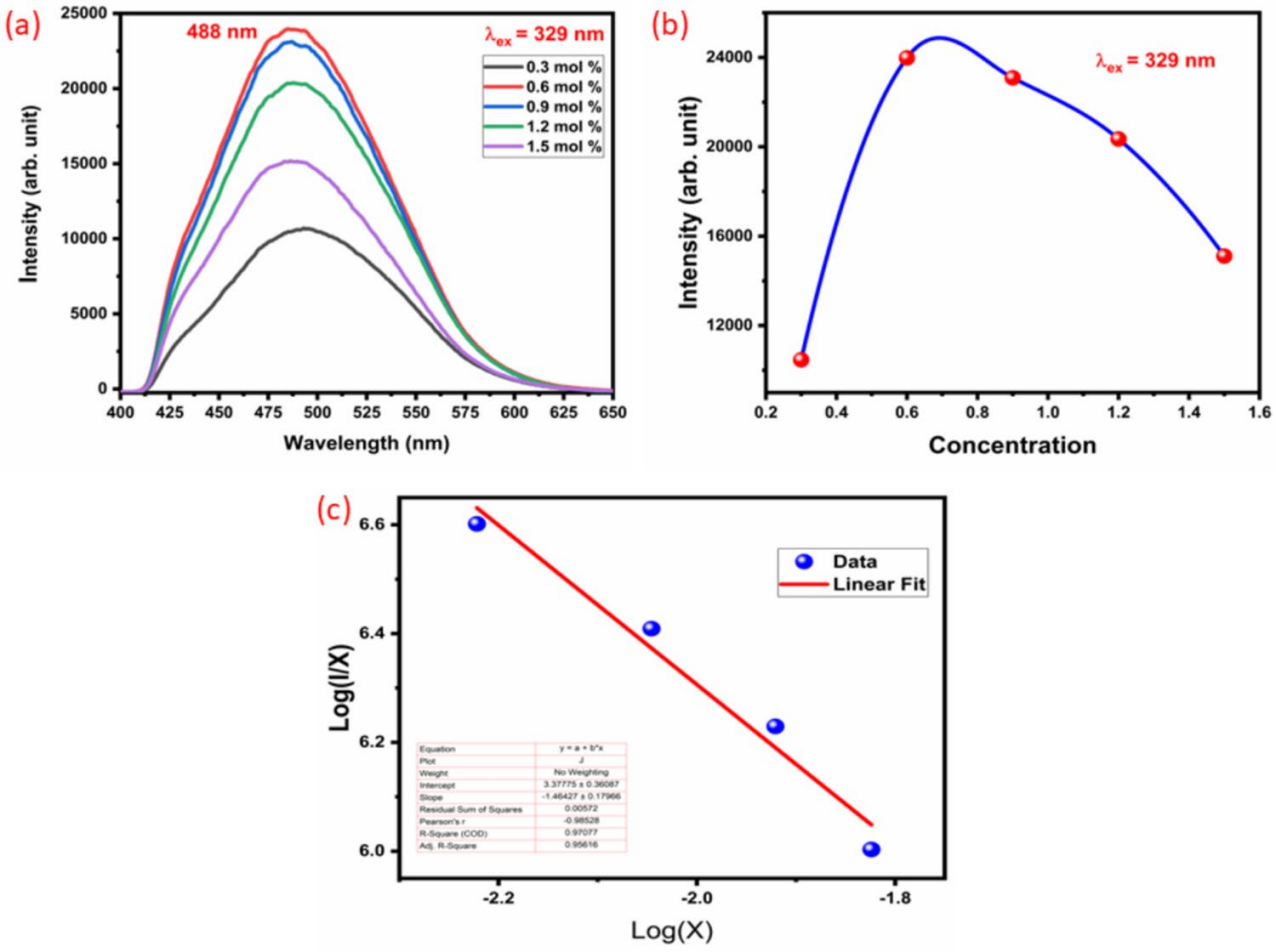
Emission spectra, concentration quenching, and Log(I/X) vs Log(X) of Y₂O₃: Bi/Cs at excitation wavelength 329 nm.

Emission spectra of Y₂O₃: Bi/Cs are shown in Fig. 25(a) at excitation wavelength 329 nm. Emission spectra are recorded for different concentrations of Cs ion from 0.3 mol% to 1.5 mol% while keeping the concentration of Bi ion at 0.3 mol% constant. Emission spectra have broad spectra from 413 nm to 625 nm cemented at 488 nm. The primary source of this emission is the ^3^P_1_→^1^S_0_ transition of Bi^3^⁺ ions, with additional contributions from charge-transfer and defect-related recombination. Site-splitting and local crystal-field variations result from Bi^3^⁺ occupying several nonequivalent Y^3^⁺ sites (two major and up to three local environments) in the cubic Y₂O₃ lattice, which is responsible for the broad nature of the spectrum. We get the same emission as Y₂O₃: Bi, which confirms the emission spectra are not affected by the doping of Cs ion. It only effect the emission intensity of PL emission. As we increase the concentration from 0.3 mol% to 0.6 mol%, PL emission intensity increases regularly, after that the PL emission intensity decreases continuously, as shown in Fig. 25(b). This is due to concentration quenching. To find the critical distance, the value of V= 1193.915 Å^3^, N=1, and X_c_ = 0.6. The calculated critical distance is 15.60802386 Å. The value of critical distance is greater than 5 Å so energy transfer in Bi ion is due to multipolar interaction. Graph of Log(I/X) vs Log(X) of Y₂O₃: Bi/Cs is shown in Fig. 25(c). By Dexter’s theory, the value of slope factor is 1.4. The value of θ is 4.2, which is near to 4. So concentration quenching is due exchange interaction.

**Fig. 26:**
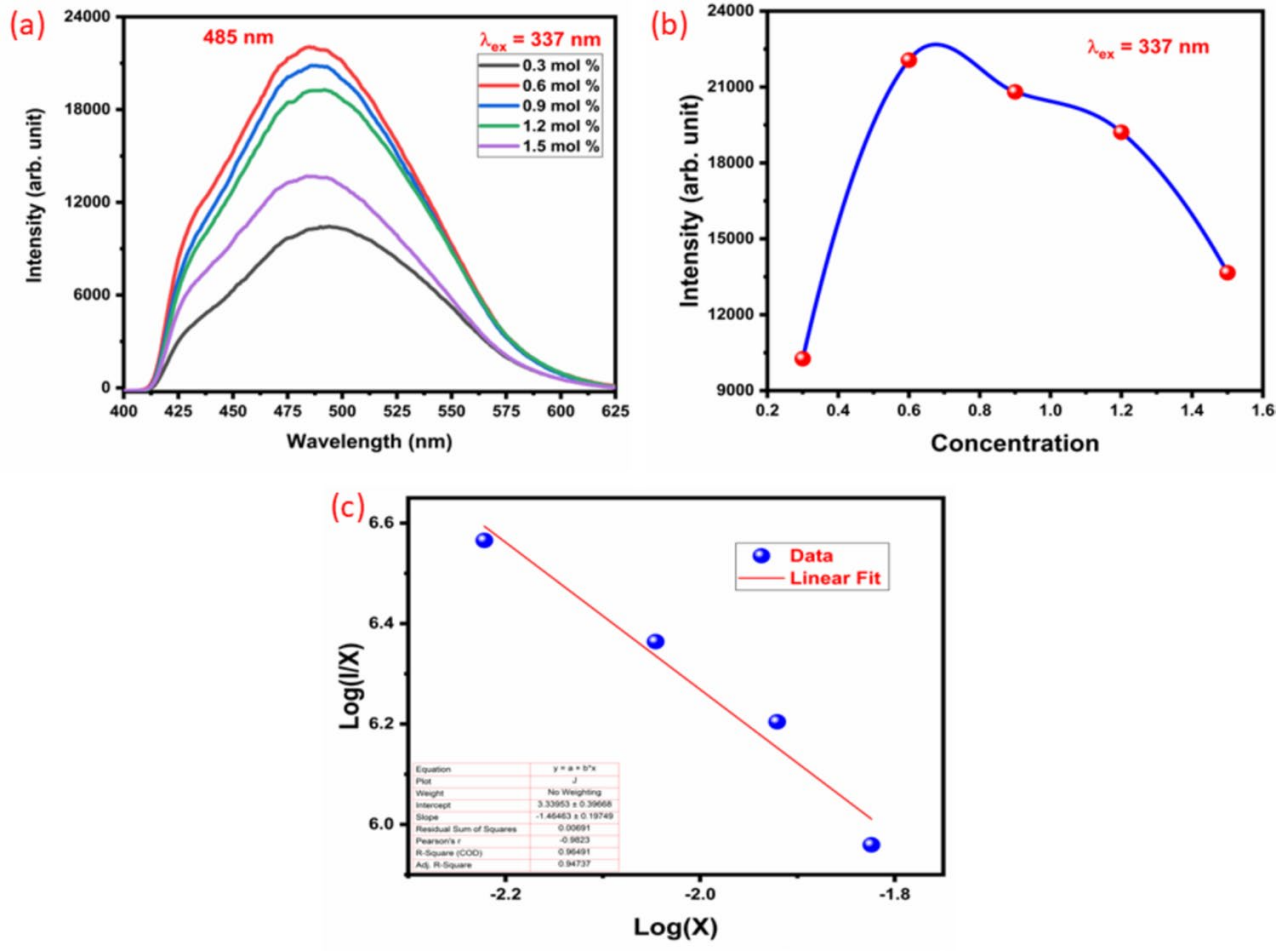
Emission spectra, concentration quenching, and Log(I/X) vs Log(X) of Y₂O₃: Bi/Cs at excitation wavelength 337 nm.

Emission spectra of Y₂O₃: Bi/Cs at excitation wavelength 337 nm are shown in Fig. 26(a). Emission spectra of Y₂O₃: Bi/Cs contain a broad spectrum from 415 nm to 625 nm centred at 485 nm, with humped near 430 nm. The ^3^P_1_→^1^S_0_ transition is represented by a similar wide emission (400–650 nm, centred at 488 nm) in Bi^3^⁺-doped Y₂O₃ under 337 nm illumination. Bi^3^⁺ ions occupying positions of the same symmetry but undergoing small changes in local crystal-field environments within the Y₂O₃ lattice cause a slight hump to develop about 430 nm. Maximum intensity is at 0.6 mol% as shown in Fig. 26(b). To find critical distance To find the critical distance , the value of V= 1193.915 Å^3^, N=1, and X_c_ = 0.6. The calculated critical distance is 15.60802386 Å. The value of critical distance is greater than 5 Å so energy transfer in Bi ion is due to multipolar interaction. Graph of Log(I/X) vs Log(X) of Y₂O₃: Bi/Cs is shown in Fig. 26(c). By Dexter’s theory, the value of slope factor is 1.46. The value of θ is 4.38, which is near to 4. So concentration quenching is due exchange interaction.

**Fig. 27:**
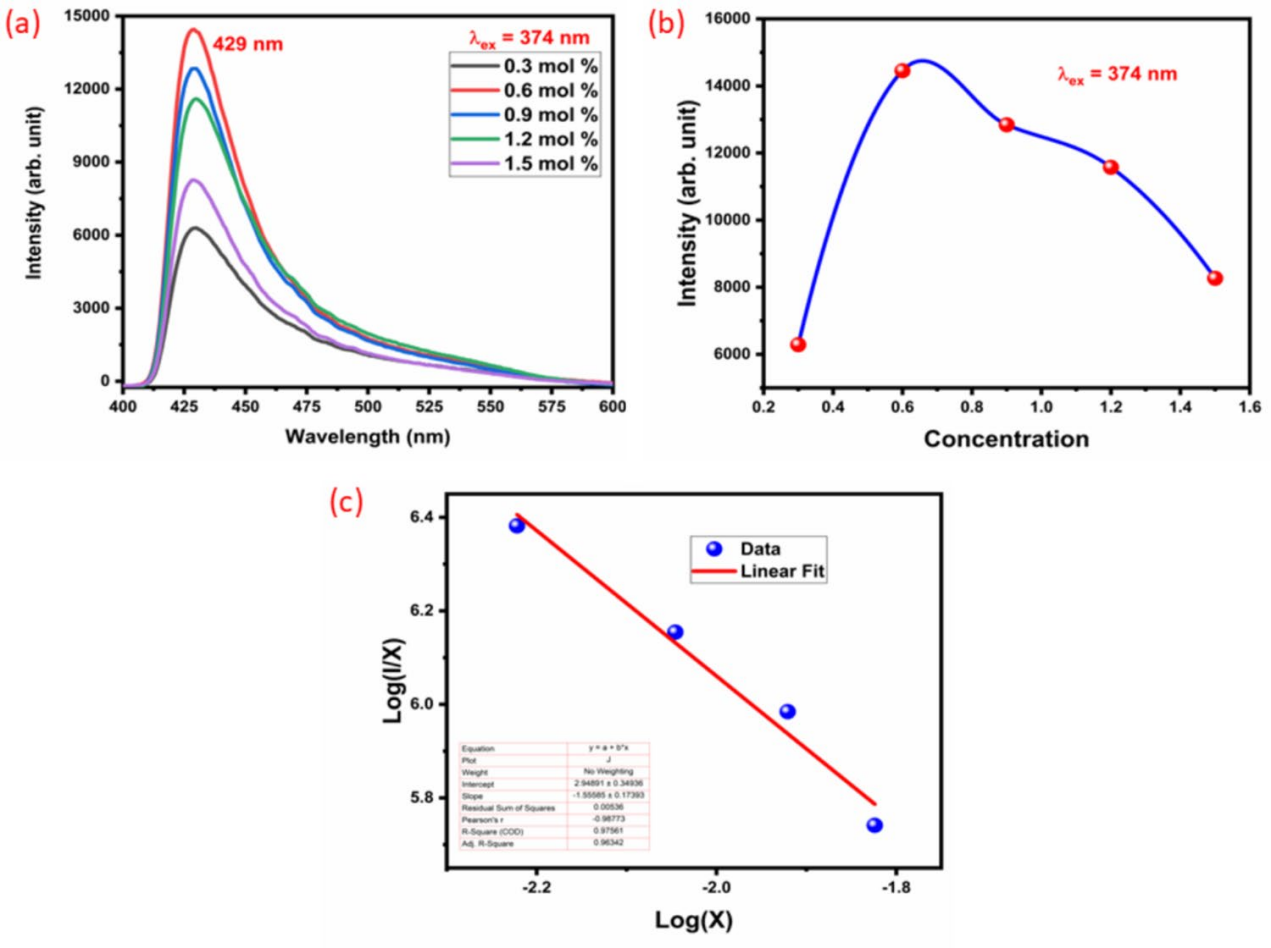
Emission spectra, concentration quenching, and Log(I/X) vs Log(X) of Y₂O₃: Bi/Cs at excitation wavelength 374 nm.

Emission spectra of Y₂O₃: Bi/Cs at excitation wavelength 374 nm are shown in Fig. 27(a). Emission spectra are centered at 429 nm due to spin-forbidden but partially permitted by high spin-orbit coupling; the corresponding ^3^P_1_→^1^S_0_ radiative decay is what is seen at 428 nm. The Y^3^⁺ sites of the cubic bixbyite lattice (mostly C₂ and C₃ᵢ symmetries) are where the emitting Bi^3^⁺ ions reside. Site-splitting, local crystal-field fluctuations (phonon sidebands), and potential neighbouring defect/CT contributions are responsible for tiny shifts or shoulders in the band. The emission of this peak is in the intense blue region. Concentration quenching happens at 0.6 mol% concentration as shown in Fig. 27(b). To find the critical distance, the value of V= 1193.915Å^3^, N=1, and X_c_ = 0.6. The calculated critical distance is 15.60802386 Å. The value of critical distance is greater than 5 Å so energy transfer in Bi ion is due to multipolar interaction. Graph of Log(I/X) vs Log(X) of Y₂O₃: Bi/Cs is shown in Fig. 27(c). By Dexter’s theory, the value of slope factor is 1.55. The value of θ is 4.65, which is near to 4. So concentration quenching is due exchange interaction.

**Fig. 28:**
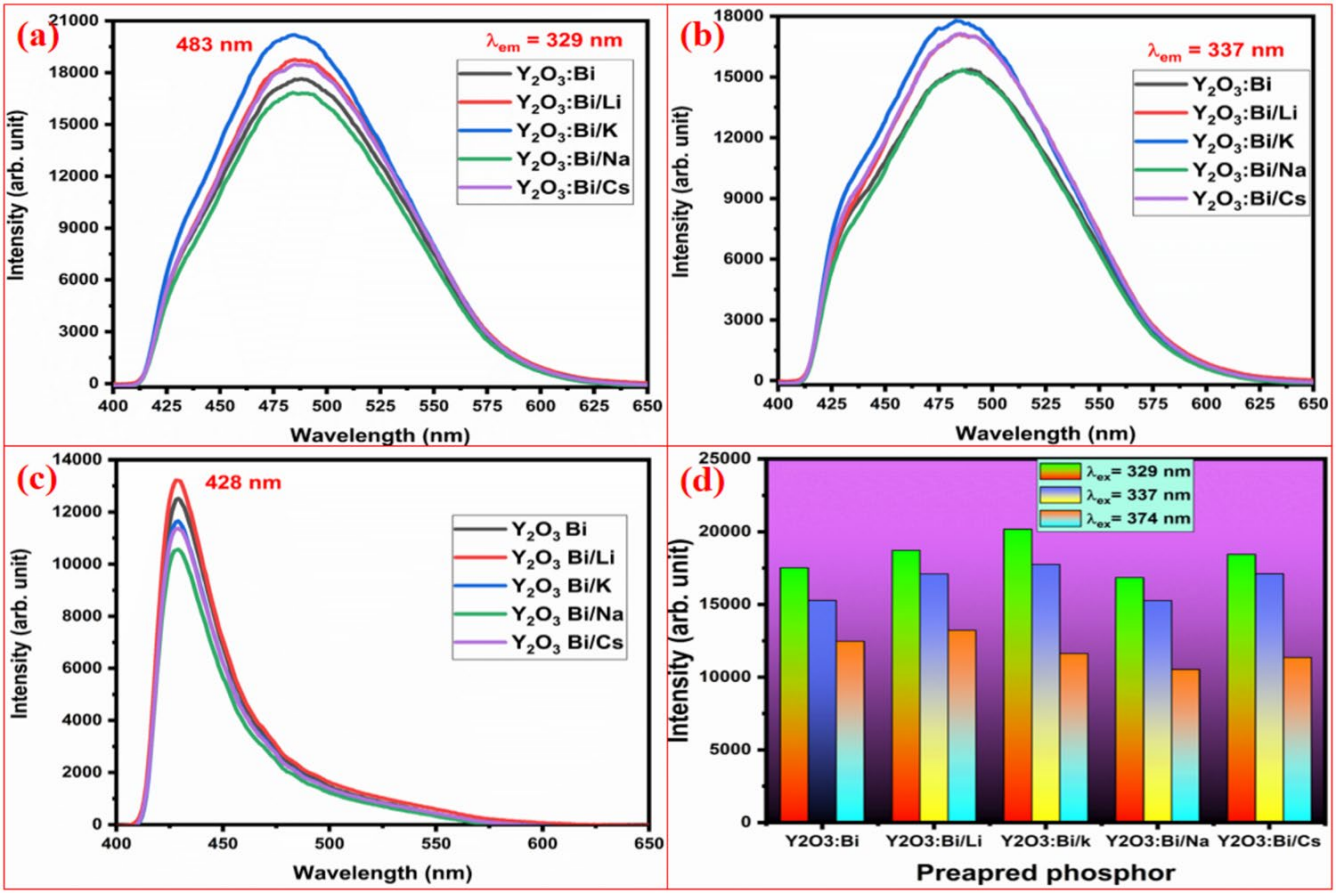
Comparing PL emission intensity of Y₂O₃ , Y₂O₃: Bi, Y₂O₃: Bi/Li, Y₂O₃: Bi/Na, Y₂O₃: Bi/K, and Y₂O₃: Bi/Cs at excitation wavelengths 329 nm, 337 nm, and 374 nm.

Figure 28 (a), (b), and (c) show the variation of PL emission intensity of Y₂O₃, Y₂O₃: Bi, Y₂O₃ : Bi/Li, Y₂O₃: Bi/Na, Y₂O₃: Bi/K, and Y₂O₃: Bi/Cs at excitation wavelengths 329 nm, 337 nm, and 374 nm, respectively. We take the concentration of each phosphor that has maximum PL intensity in that corresponding series. The order of PL emission intensity is Y₂O₃: Bi/K > Y₂O₃: Bi/Li > Y₂O₃: Bi/Cs > Y₂O₃: Bi > Y₂O₃: Bi/Na for emission spectra excited by 329 nm. Order for PL emission spectra when it is excited by 337 nm is Y₂O₃: Bi/K > Y₂O₃: Bi/Cs > Y₂O₃: Bi/Na ≈ Y₂O₃: Bi/Li ≈ Y₂O₃: Bi, while for phosphor when it is excited by 374 nm Y₂O₃: Bi/Li > Y₂O₃: Bi > Y₂O₃: Bi/K > Y₂O₃: Bi/Cs > Y₂O₃: Bi/Na. The Fig. 28(d) shows the variation of intensity with respect to different phosphors in a bar graph. Y₂O₃: Bi/K, Y₂O₃: Bi/K, and Y₂O₃: Bi/Li emit maximum PL emission intensity at different excitation wavelengths 329 nm, 337 nm, and 374 nm, respectively.

### CIE

Commission Internationale de l’´ Eclairage (CIE) is used to calculate the CIE coordinate of emission phosphor. By using the CIE coordinate, we can find the color purity of the prepared phosphor. Colour purity of phosphor can be given by :

3 $$Color{ }purity = \frac{{\sqrt {\left( {X - X_{i} } \right)^{2} + \left( {Y - Y_{i} } \right)^{2} } }}{{\sqrt {\left( {X_{d} - X_{i} } \right)^{2} + \left( {Y_{d} - Y_{i} } \right)^{2} } }}{*}100{\text{ \% }}$$

Where (x,y) are calculated CIE co-ordinate using CIE software, (x_d_, y_d_) are the CIE co-ordinate of the dominant wavelength, and (x_i_,y_i_) are co-ordinate of white light^32^.

#### Y₂O₃: Bi

**Fig. 29:**
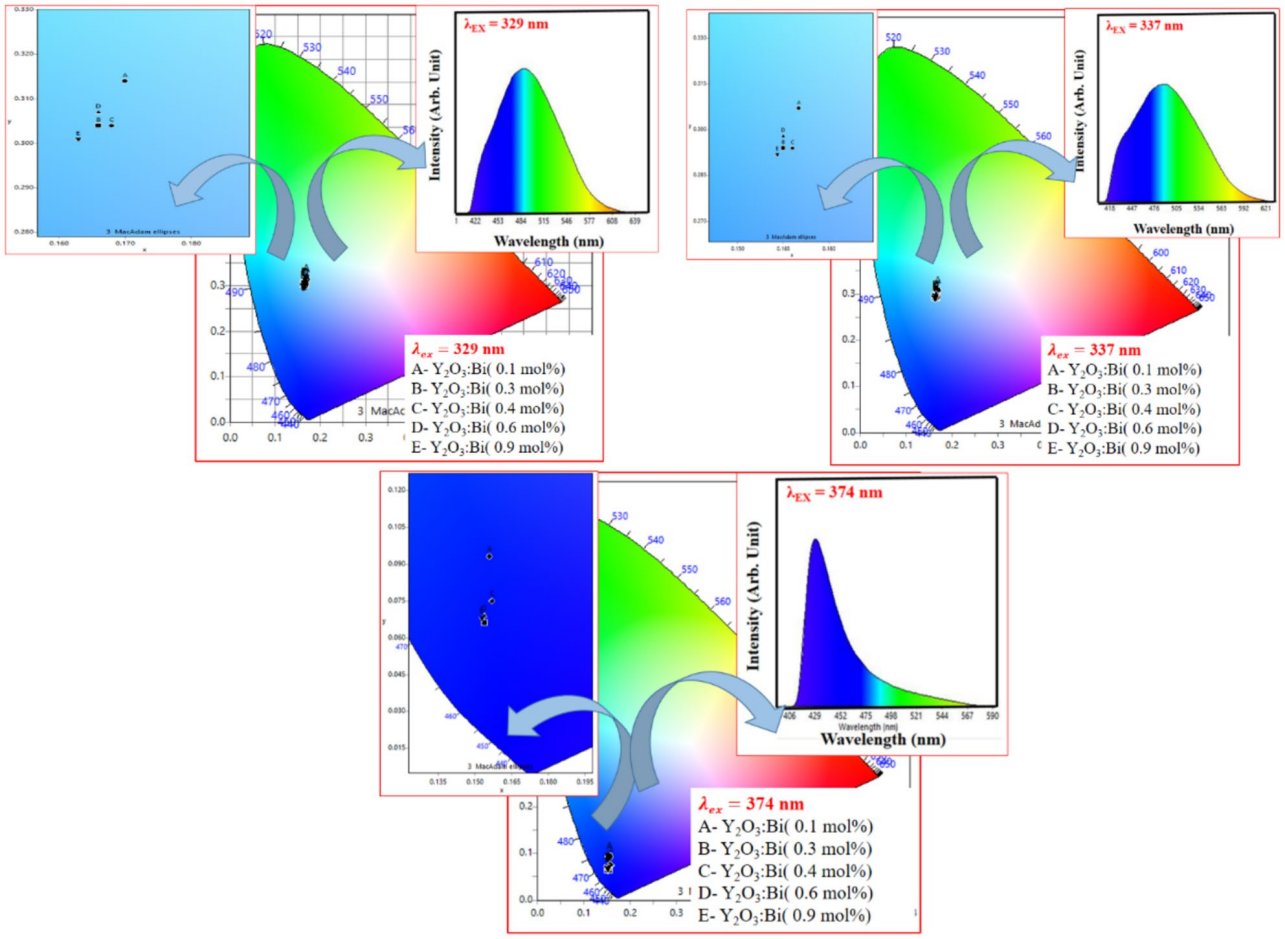
CIE diagram of Y₂O₃: Bi at excitation wavelength 329, 337 & 374 nm.

Figure 29(a), (b) and (c) show the CIE diagram of Y₂O₃: Bi at excitation wavelengths of 329 nm, 337 nm, and 374 nm, respectively. Cie co-ordinate of Y₂O₃: Bi at excitation wavelengths of 329 nm, 337 nm are near the white region. Comparing between excitation wavelength 329 nm and 337 nm CIE co-ordinate of excitation at 329 is slightly toward the green region. CIE co-ordinate of Y₂O₃: Bi at the excitation wavelength are in the deep blue region. This phosphor can be useful for blue LEDs applications. While phosphors excited at 329 nm, and 337 nm can be useful in WLEDs applications. The detailed CIE coordinates, dominant wavelength, and color purity values for Y₂O₃: Bi excited at 329 nm, 337 nm, and 374 nm are presented in the Tables 6, 7, and 8, respectively.

**Table 6:** CIE co-ordinate, color purity of Y₂O₃: Bi at excitation wavelength of 329 nm.

Y₂O₃: Bi at excitation wavelength 329 nm						
SR	Name	X	Y	Xi	Yi	Color purity
1	Y₂O₃: Bi( 0.1 mol%)	0.170	0.314	0.073	0.185	51.70
2	Y₂O₃: Bi( 0.3 mol%)	0.166	0.304	0.064	0.217	54.50
3	Y₂O₃: Bi( 0.4 mol%)	0.168	0.304	0.055	0.254	54.31
4	Y₂O₃: Bi( 0.6 mol%)	0.166	0.307	0.050	0.274	54.79
5	Y₂O₃: Bi( 0.9 mol%)	0.163	0.301	0.069	0.201	55.34

**Table 7:** CIE co-ordinate, color purity of Y₂O₃: Bi at excitation wavelength of 337 nm.

Y₂O₃: Bi at excitation wavelength 337 nm						
SR	Name	X	Y	Xi	Yi	Color purity
1	Y₂O₃: Bi( 0.1 mol%)	0.170	0.307	0.059	0.235	53.20
2	Y₂O₃: Bi( 0.3 mol%)	0.165	0.294	0.059	0.235	55.62
3	Y₂O₃: Bi( 0.4 mol%)	0.168	0.294	0.059	0.235	54.49
4	Y₂O₃: Bi( 0.6 mol%)	0.165	0.298	0.069	0.201	54.72
5	Y₂O₃: Bi( 0.9 mol%)	0.163	0.292	0.064	0.217	56.18

**Table 8:** CIE co-ordinate, color purity of Y₂O₃: Bi at excitation wavelength of 374 nm.

Y₂O₃: Bi at excitation wavelength 374 nm						
SR	Name	X	Y	Xi	Yi	Color purity
1	Y₂O₃: Bi( 0.1 mol%)	0.156	0.093	0.169	0.007	79.80
2	Y₂O₃: Bi( 0.3 mol%)	0.154	0.066	0.169	0.007	86.77
3	Y₂O₃: Bi( 0.4 mol%)	0.157	0.075	0.169	0.007	84.05
4	Y₂O₃: Bi( 0.6 mol%)	0.154	0.069	0.169	0.007	86.02
5	Y₂O₃: Bi( 0.9 mol%)	0.153	0.068	0.169	0.007	86.42

#### Y₂O₃: Bi/Li

**Fig. 30:**
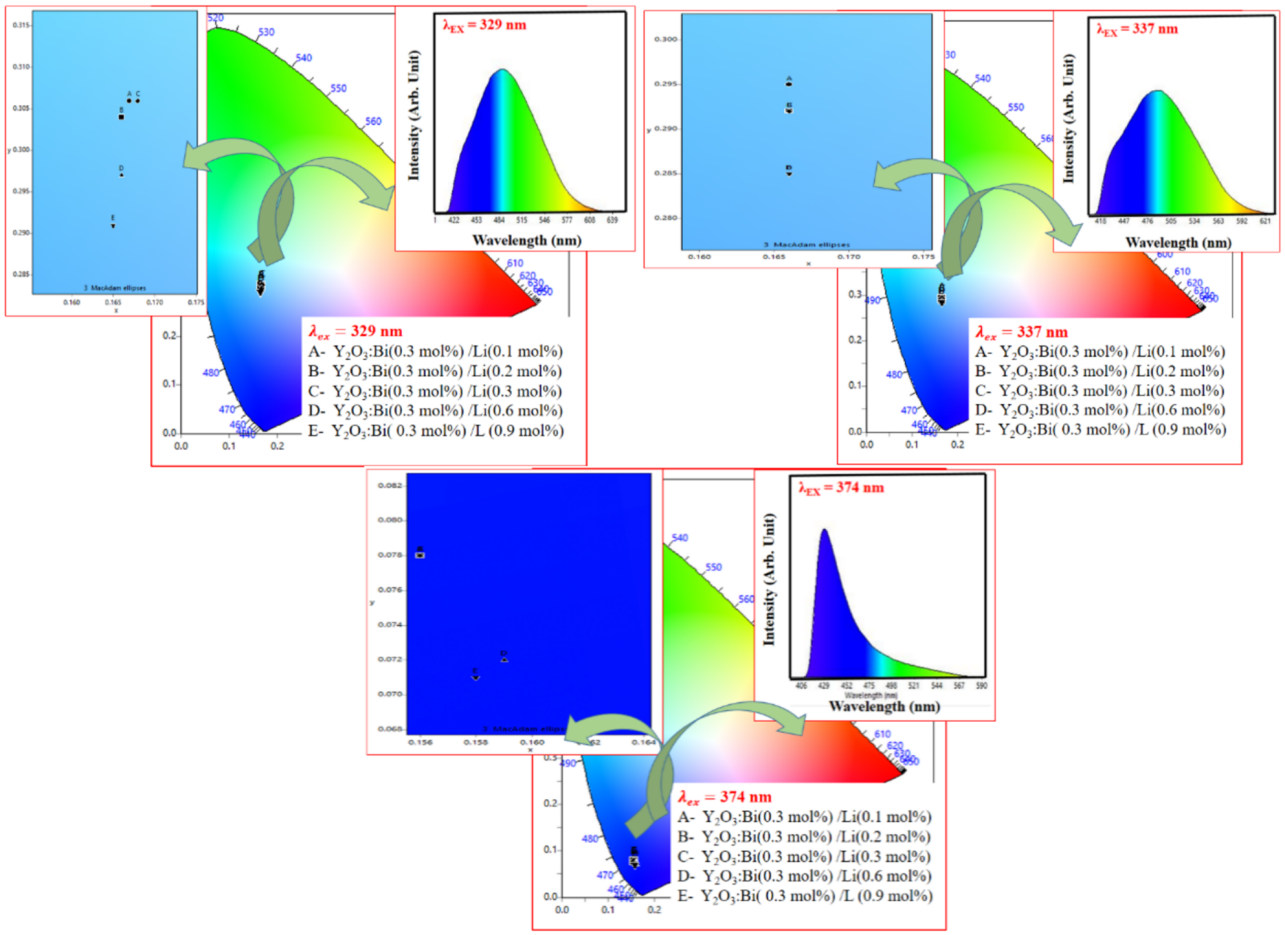
CIE diagram of Y₂O₃: Bi/Li at excitation wavelength 329, 337 & 374 nm.

The CIE diagram of Y₂O₃: Bi/Li at excitation wavelengths of 329 nm, 337 nm, and 374 nm is displayed in Fig. 30(a), (b) and (c), respectively. Y₂O₃: Bi/Li Cie coordinates at excitation wavelengths of 329 and 337 nm are close to the white area. When comparing the excitation wavelengths of 329 and 337 nm, the excitation coordinate at 329 nm is slightly in the green region. At the excitation wavelength, Y₂O₃: Bi/Li CIE coordinate is in the deep blue zone. The application of blue LEDs may benefit from this phosphor. In WLED applications, phosphor stimulated at 329 and 337 nm can be beneficial. Tables 9, 10, and 11 show the precise CIE coordinates, dominant wavelength, and color purity values for Y₂O₃: Bi/Li stimulated at 329 nm, 337 nm, and 374 nm, respectively.

**Table 9:** CIE co-ordinate, color purity of Y₂O₃: Bi/Li at excitation wavelength of 329 nm.

Y₂O₃: Bi / Li at excitation wavelength 329 nm						
SR	Name	X	Y	Xi	Yi	Color purity
1	Y₂O₃: Bi( 0.3 mol%)/Li(0.1 mol%)	0.167	0.306	0.059	0.235	54.36
2	Y₂O₃: Bi( 0.3 mol%)/Li(0.2 mol%)	0.166	0.304	0.059	0.235	54.79
3	Y₂O₃: Bi( 0.3 mol%)/Li(0.3 mol%)	0.168	0.306	0.059	0.235	53.98
4	Y₂O₃: Bi( 0.3 mol%)/Li(0.6 mol%)	0.166	0.297	0.059	0.235	55.08
5	Y₂O₃: Bi( 0.3 mol%)//Li(0.9 mol%)	0.165	0.291	0.073	0.185	54.34

**Table 10:** CIE co-ordinate, color purity of Y₂O₃: Bi/Li at excitation wavelength of 337 nm.

Y₂O₃: Bi / Li at excitation wavelength 337 nm						
SR	Name	X	Y	Xi	Yi	Color purity
1	Y₂O₃: Bi( 0.3 mol%)/Li(0.1 mol%)	0.166	0.295	0.059	0.235	55.18
2	Y₂O₃: Bi( 0.3 mol%)/Li(0.2 mol%)	0.166	0.292	0.064	0.217	55.06
3	Y₂O₃: Bi( 0.3 mol%)/Li(0.3 mol%)	0.166	0.292	0.064	0.217	54.03
4	Y₂O₃: Bi( 0.3 mol%)/Li(0.6 mol%)	0.166	0.285	0.069	0.201	55.17
5	Y₂O₃: Bi( 0.3 mol%)//Li(0.9 mol%)	0.166	0.285	0.069	0.201	54.69

**Table 11:** CIE co-ordinate, color purity of Y₂O₃: Bi/Li at excitation wavelength of 374 nm.

Y₂O₃: Bi / Li at excitation wavelength 374 nm						
SR	Name	X	Y	Xi	Yi	Color purity
1	Y₂O₃: Bi( 0.3 mol%)/Li(0.1 mol%)	0.156	0.078	0.169	0.007	83.47
2	Y₂O₃: Bi( 0.3 mol%)/Li(0.2 mol%)	0.156	0.078	0.169	0.007	85.46
3	Y₂O₃: Bi( 0.3 mol%)/Li(0.3 mol%)	0.156	0.078	0.169	0.007	82.87
4	Y₂O₃: Bi( 0.3 mol%)/Li(0.6 mol%)	0.159	0.072	0.170	0.006	84.37
5	Y₂O₃: Bi( 0.3 mol%)//Li(0.9 mol%)	0.158	0.071	0.170	0.006	84.77

#### Y₂O₃: Bi/Na

**Fig. 31:**
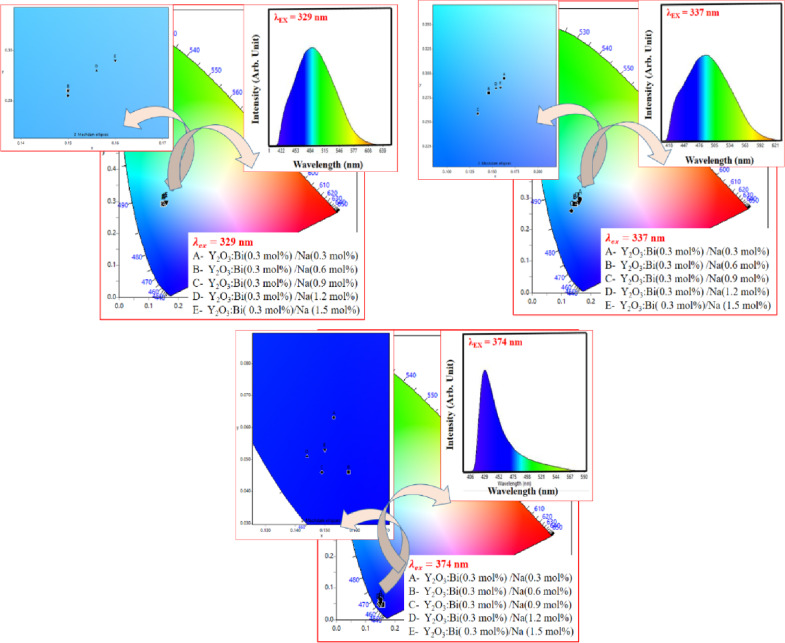
CIE diagram of Y₂O₃: Bi/Na at excitation wavelength 329 nm.

The CIE diagram of Y₂O₃: Bi/Na at excitation wavelengths of 329 nm, 337 nm, and 374 nm is shown in Fig. 31(a), (b) and (c), respectively. At excitation wavelengths of 329 and 337 nm, the CIE coordinate of Y₂O₃: Bi is close to the white region. When comparing the excitation wavelengths of 329 nm and 337 nm, the excitation coordinate at 329 nm is marginally closer to the green region. At the excitation wavelength, the CIE coordinate of Y₂O₃: Bi/Na is in the deep blue zone. This phosphor has potential use in blue LEDs. In WLED applications, phosphor stimulated at 329 nm and 337 nm can be helpful. Tables 12, 13, and 14 show the color purity values, dominating wavelength, and precise CIE coordinates for Y₂O₃: Bi/Na stimulated at 329 nm, 337 nm, and 374 nm, respectively.

**Table 12:** CIE co-ordinate, color purity of Y₂O₃: Bi/Na at excitation wavelength of 329 nm.

Y₂O₃: Bi / Na at excitation wavelength 329 nm						
SR	Name	X	Y	Xi	Yi	Color purity
1	Y₂O₃: Bi( 0.3 mol%)/Na(0.3 mol%)	0.150	0.291	0.109	0.087	54.44
2	Y₂O₃: Bi( 0.3 mol%)/Na(0.6 mol%)	0.150	0.292	0.110	0.087	53.21
3	Y₂O₃: Bi( 0.3 mol%)/Na(0.9 mol%)	0.150	0.292	0.110	0.087	56.46
4	Y₂O₃: Bi( 0.3 mol%)/Na(1.2 mol%)	0.156	0.296	0.078	0.170	56.65
5	Y₂O₃: Bi( 0.3 mol%)//Na(1.5mol%)	0.160	0.298	0.064	0.217	56.98

**Table 13:** CIE co-ordinate, color purity of Y₂O₃: Bi/ Na at excitation wavelength of 337 nm.

Y₂O₃: Bi / Na at excitation wavelength 337 nm						
SR	Name	X	Y	Xi	Yi	Color purity
1	Y₂O₃: Bi( 0.3 mol%)/Na(0.3 mol%)	0.163	0.295	0.064	0.217	56
2	Y₂O₃: Bi( 0.3 mol%)/Na(0.6 mol%)	0.146	0.280	0.113	0.080	54.62
3	Y₂O₃: Bi( 0.3 mol%)/Na(0.9 mol%)	0.134	0.259	0.113	0.080	60.18
4	Y₂O₃: Bi( 0.3 mol%)/Na(1.2 mol%)	0.154	0.285	0.073	0.185	58.74
5	Y₂O₃: Bi( 0.3 mol%)//Na(1.5mol%)	0.159	0.286	0.069	0.201	57.66

**Table 14:** CIE co-ordinate, color purity of Y₂O₃: Bi/ Na at excitation wavelength of 374 nm.

Y₂O₃: Bi / Na at excitation wavelength 374 nm						
SR	Name	X	Y	Xi	Yi	Color purity
1	Y₂O₃: Bi( 0.3 mol%)/Na(0.3 mol%)	0.153	0.063	0.170	0.006	87.54
2	Y₂O₃: Bi( 0.3 mol%)/Na(0.6 mol%)	0.158	0.046	0.169	0.007	98.40
3	Y₂O₃: Bi( 0.3 mol%)/Na(0.9 mol%)	0.149	0.046	0.165	0.010	99
4	Y₂O₃: Bi( 0.3 mol%)/Na(1.2 mol%)	0.144	0.051	0.169	0.007	93.99
5	Y₂O₃: Bi( 0.3 mol%)//Na(1.5mol%)	0.150	0.053	0.170	0.006	90.51

#### Y₂O₃: Bi/K

**Fig. 32:**
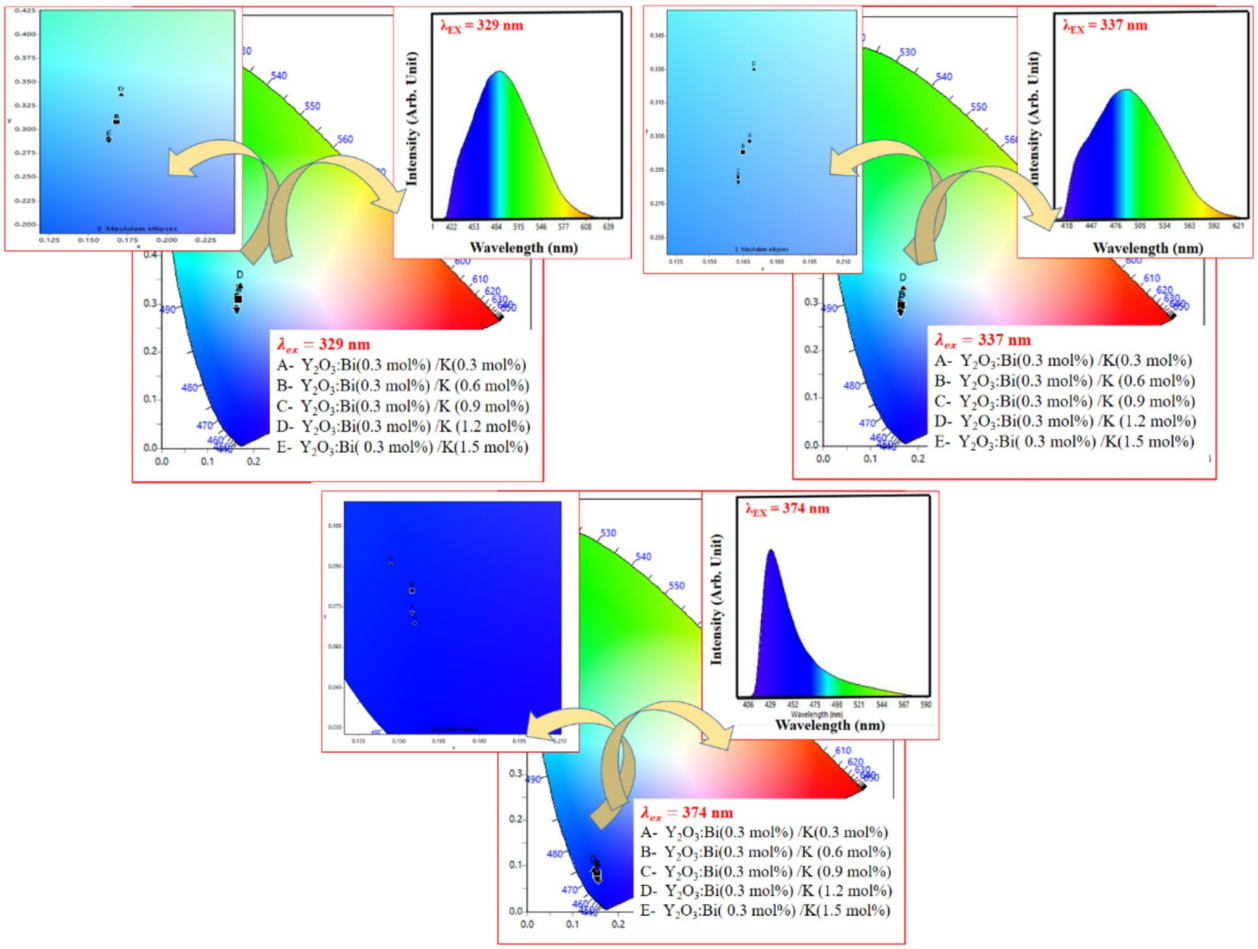
CIE diagram of Y₂O₃: Bi/K at excitation wavelength 329, 337 & 374 nm.

The CIE diagram of Y₂O₃: Bi/K at excitation wavelengths of 329 nm, 337 nm, and 374 nm is depicted in Fig. 32(a), (b) and (c), respectively. At excitation wavelengths of 329 nm and 337 nm, the Cie coordinates of Y₂O₃: Bi/K are close to the white region. When comparing the excitation wavelengths of 329 and 337 nm, the excitation coordinate at 329 nm is marginally closer to the green region. Y₂O₃: Bi/K CIE coordinate at the excitation wavelength is in the deep blue area. The usage of this phosphor in blue LED applications may be beneficial. In WLED applications, phosphor stimulated at 329 and 337 nm can be helpful. Tables 15, 16, and 17 display the specific CIE coordinates, dominating wavelength, and color purity values for Y₂O₃: Bi/K excited at 329 nm, 337 nm, and 374 nm, respectively.

**Table 15:** CIE co-ordinate, color purity of Y₂O₃: Bi/K at excitation wavelength of 329 nm.

Y₂O₃: Bi / K at excitation wavelength 329 nm						
SR	Name	X	Y	Xi	Yi	Color purity
1	Y₂O₃: Bi( 0.3 mol%)/K(0.3 mol%)	0.168	0.308	0.059	0.235	53.93
2	Y₂O₃: Bi( 0.3 mol%)/K(0.6 mol%)	0.168	0.308	0.170	0.165	53.11
3	Y₂O₃: Bi( 0.3 mol%)/K(0.9 mol%)	0.163	0.291	0.073	0.185	55.07
4	Y₂O₃: Bi( 0.3 mol%)/K(1.2 mol%)	0.171	0.337	0.027	0.388	48.15
5	Y₂O₃: Bi( 0.3 mol%)/K(1.5mol%)	0.163	0.289	0.027	0.388	51.21

**Table 16:** CIE co-ordinate, color purity of Y₂O₃: Bi/K at excitation wavelength of 337 nm.

Y₂O₃: Bi / K at excitation wavelength 337 nm						
SR	Name	X	Y	Xi	Yi	Color purity
1	Y₂O₃: Bi( 0.3 mol%)/K(0.3 mol%)	0.168	0.298	0.059	0.235	54.28
2	Y₂O₃: Bi( 0.3 mol%)/K(0.6 mol%)	0.165	0.293	0.069	0.201	54.99
3	Y₂O₃: Bi( 0.3 mol%)/K(0.9 mol%)	0.163	0.282	0.073	0.185	55.73
4	Y₂O₃: Bi( 0.3 mol%)/K(1.2 mol%)	0.170	0.330	0.036	0.340	51.16
5	Y₂O₃: Bi( 0.3 mol%)/K(1.5mol%)	0.163	0.280	0.078	0.170	55.22

**Table 17:** CIE co-ordinate, color purity of Y₂O₃: Bi/K at excitation wavelength of 374 nm.

Y₂O₃: Bi / K at excitation wavelength 374 nm						
SR	Name	X	Y	Xi	Yi	Color purity
1	Y₂O₃: Bi( 0.3 mol%)/K(0.3 mol%)	0.155	0.081	0.169	0.007	82.89
2	Y₂O₃: Bi( 0.3 mol%)/K(0.6 mol%)	0.155	0.081	0.169	0.007	85.36
3	Y₂O₃: Bi( 0.3 mol%)/K(0.9 mol%)	0.156	0.069	0.169	0.007	85.71
4	Y₂O₃: Bi( 0.3 mol%)/K(1.2 mol%)	0.147	0.091	0.169	0.007	81.81
5	Y₂O₃: Bi( 0.3 mol%)/K(1.5mol%)	0.155	0.073	0.170	0.006	84.74

#### Y₂O₃: Bi/Cs

**Fig. 33:**
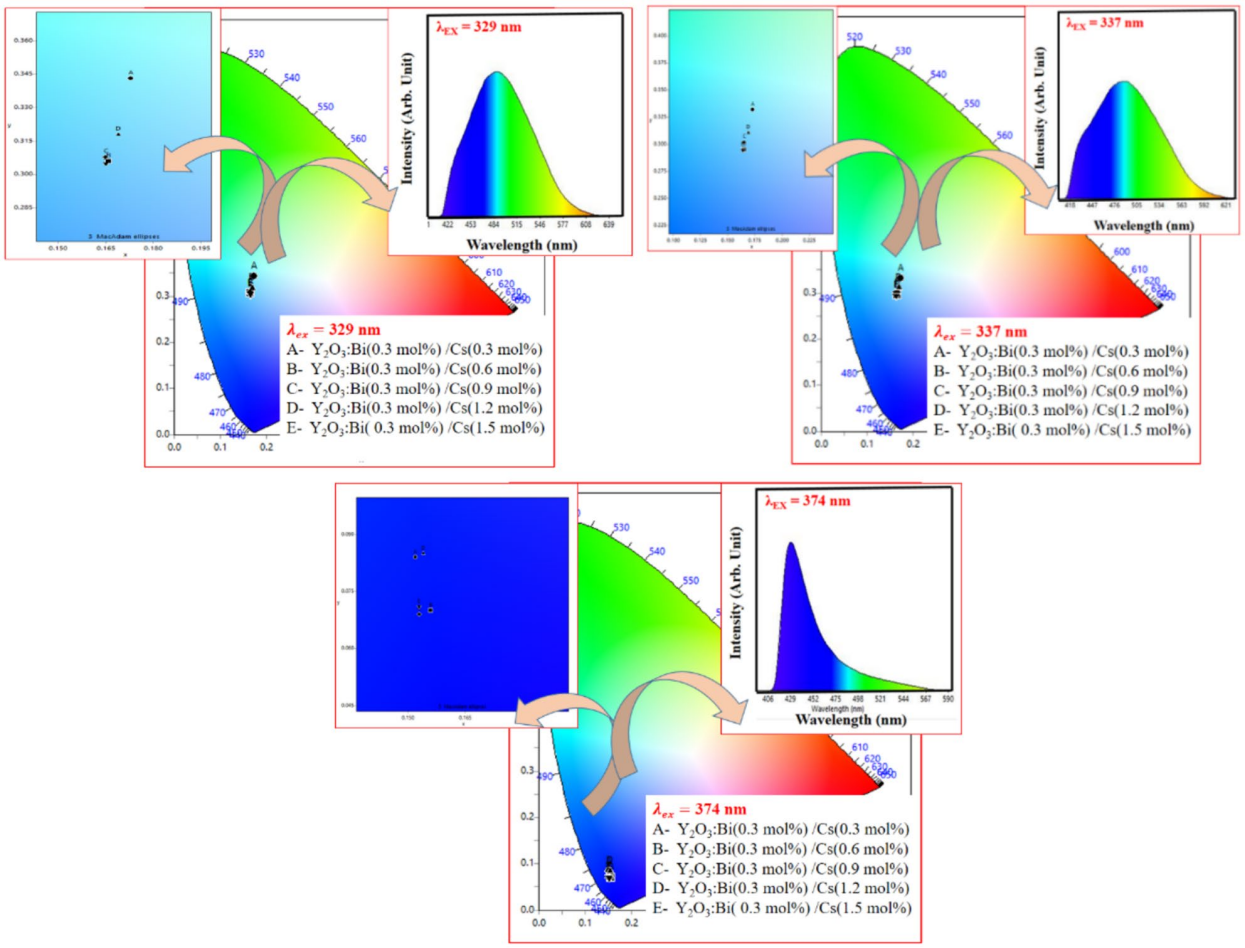
CIE diagram of Y₂O₃: Bi/Cs at excitation wavelength 329, 337 & 374 nm.

Figure 33(a), (b) and (c), show the CIE diagram of Y₂O₃: Bi/Cs at excitation wavelengths at 329 nm, 337 nm, and 374 nm, respectively. Cie co-ordinate of Y₂O₃: Bi/Cs at excitation wavelengths at 329 nm, 337 nm are near the white region. Comparing between excitation wavelength 329 nm and 337 nm CIE co-ordinate of excitation at 329 is slightly toward the green region. CIE co-ordinate of Y₂O₃: Bi/Cs at excitation wavelength is in the deep blue region. Y₂O₃: Bi/Cs(0.3 mol%) has a CIE co-ordinate in the cyan region at an excitation wavelength of 329 nm. This phosphor can be useful for blue LEDs applications. While phosphors excited at 329 nm and 337 nm can be useful in WLEDs applications. The detailed CIE coordinates, dominant wavelength, and color purity values for Y₂O₃: Bi excited at 329 nm, 337 nm, and 374 nm are presented in Tables 18, 19, and 20, respectively.

**Table 18:** CIE co-ordinate, color purity of Y₂O₃: Bi/Cs at excitation wavelength of 329 nm.

Y₂O₃: Bi / Cs at excitation wavelength 329 nm						
SR	Name	X	Y	Xi	Yi	Color purity
1	Y₂O₃: Bi( 0.3 mol%)/Cs(0.3 mol%)	0.173	0.343	0.032	0.364	49.50
2	Y₂O₃: Bi( 0.3 mol%)/Cs(0.6 mol%)	0.166	0.306	0.064	0.217	54.44
3	Y₂O₃: Bi( 0.3 mol%)/Cs(0.9 mol%)	0.165	0.308	0.064	0.217	54.77
4	Y₂O₃: Bi( 0.3 mol%)/Cs(1.2 mol%)	0.169	0.318	0.059	0.235	53.47
5	Y₂O₃: Bi( 0.3 mol%)//Cs(1.5mol%)	0.165	0.305	0.059	0.235	55.14

**Table 19:** CIE co-ordinate, color purity of Y₂O₃: Bi/Cs at excitation wavelength of 337 nm.

Y₂O₃: Bi / Cs at excitation wavelength 337 nm						
SR	Name	X	Y	Xi	Yi	Color purity
1	Y₂O₃: Bi( 0.3 mol%)/Cs(0.3 mol%)	0.173	0.332	0.045	0.295	51.89
2	Y₂O₃: Bi( 0.3 mol%)/Cs(0.6 mol%)	0.165	0.295	0.064	0.217	55.26
3	Y₂O₃: Bi( 0.3 mol%)/Cs(0.9 mol%)	0.165	0.302	0.059	0.235	55.24
4	Y₂O₃: Bi( 0.3 mol%)/Cs(1.2 mol%)	0.169	0.311	0.050	0.274	55.58
5	Y₂O₃: Bi( 0.3 mol%)//Cs(1.5mol%)	0.164	0.295	0.059	0.235	55.93

**Table 20:** CIE co-ordinate, color purity of Y₂O₃: Bi/Cs at excitation wavelength of 374 nm.

Y₂O₃: Bi / Cs at excitation wavelength 374 nm						
SR	Name	X	Y	Xi	Yi	Color purity
1	Y₂O₃: Bi( 0.3 mol%)/Cs(0.3 mol%)	0.152	0.084	0.169	0.007	82.65
2	Y₂O₃: Bi( 0.3 mol%)/Cs(0.6 mol%)	0.156	0.070	0.169	0.007	85.46
3	Y₂O₃: Bi( 0.3 mol%)/Cs(0.9 mol%)	0.153	0.069	0.169	0.007	86.18
4	Y₂O₃: Bi( 0.3 mol%)/Cs(1.2 mol%)	0.154	0.085	0.169	0.007	82.08
5	Y₂O₃: Bi( 0.3 mol%)//Cs(1.5mol%)	0.153	0.071	0.169	0.007	85.68

## Application

**Fig. 34:**
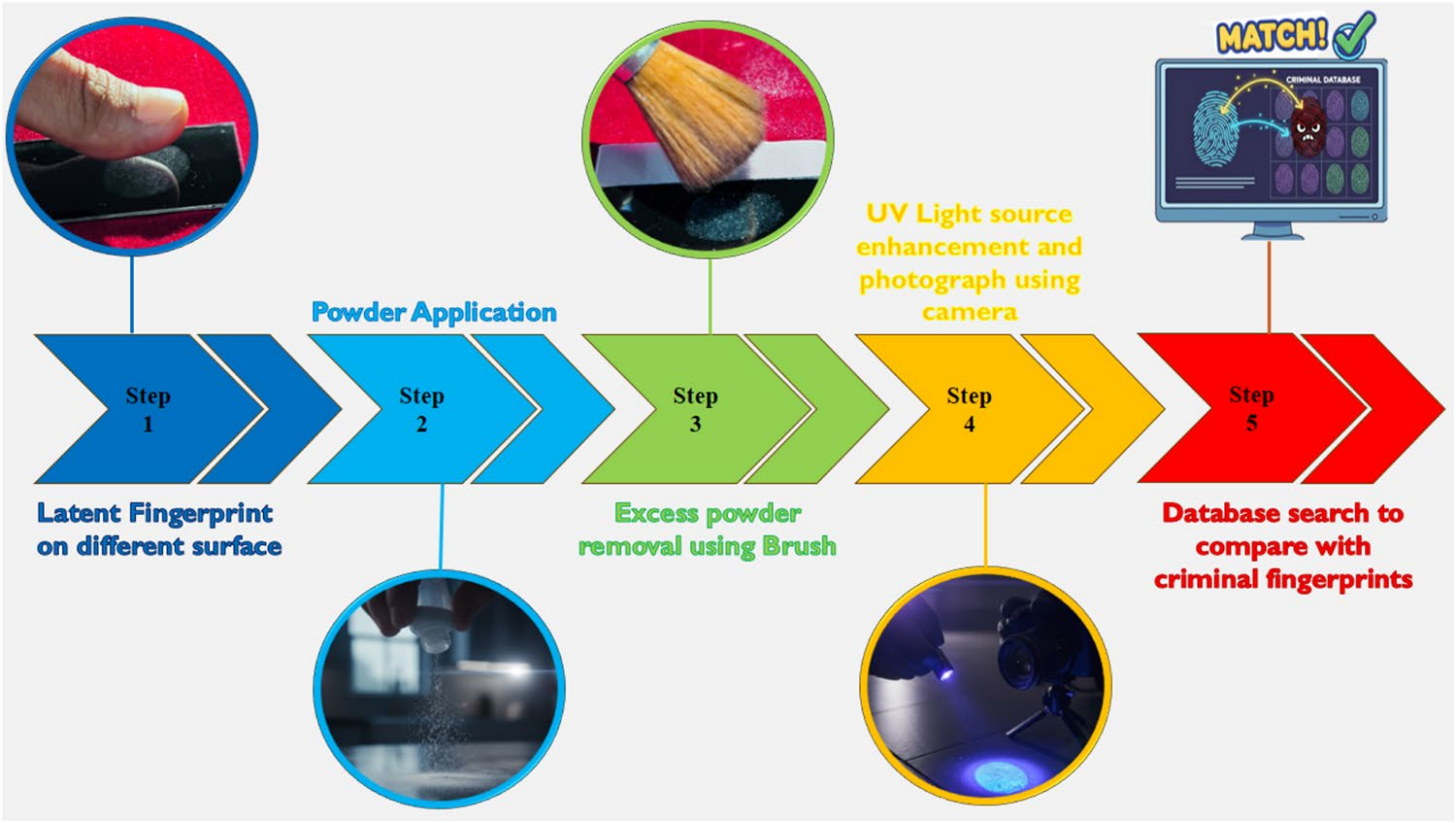
Mechanism of fingerprint detection using phosphor.

The distinctive features of epidermal ridge patterns and their spatial correlations make fingerprints unique biometric identifiers. Sweat pores range in diameter from 88 to 220 µm, and the average adult fingerprint ridge width is about 450 µm^39,40^. The latent fingerprint is initially located for phosphorescent powder forensic viewing. After that, the area is treated with a specific phosphor powder that sticks to the ridge pattern’s remaining moisture and oils. A delicate brush is used to carefully remove extra powder, leaving the material mostly on the fingerprint residues. After that, ultraviolet (UV) light is applied to the area. This high-energy light is absorbed by the phosphor, which then experiences photoluminescence and releases visible light. The pattern can be captured on camera for later examination and comparison with biometric databases thanks to this improved contrast. The accompanying Fig. 34 provides an illustration of this process^41,42^.

**Fig. 35:**
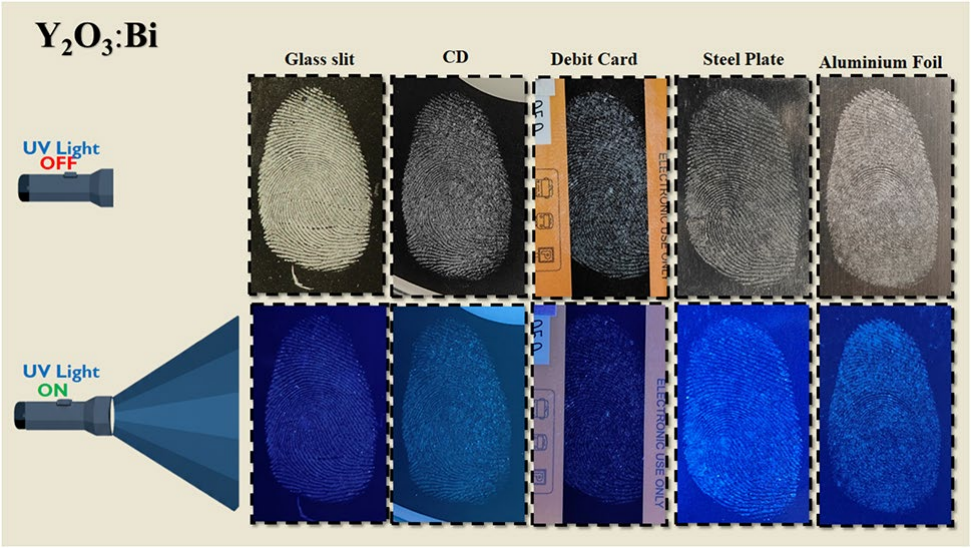
Fluorescence images of latent fingerprints stained by Y₂O₃: Bi phosphors.

**Fig. 36:**
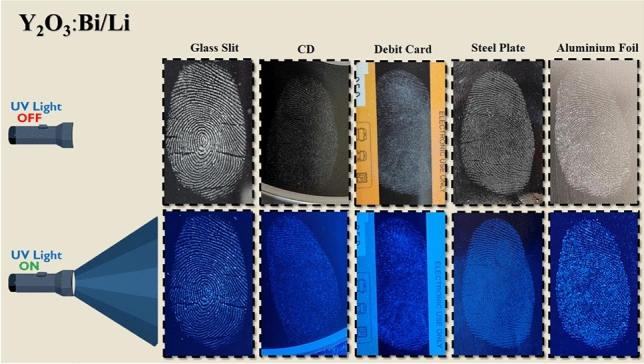
Fluorescence images of latent fingerprints stained by Y₂O₃: Bi/Li phosphors.

**Fig. 37:**
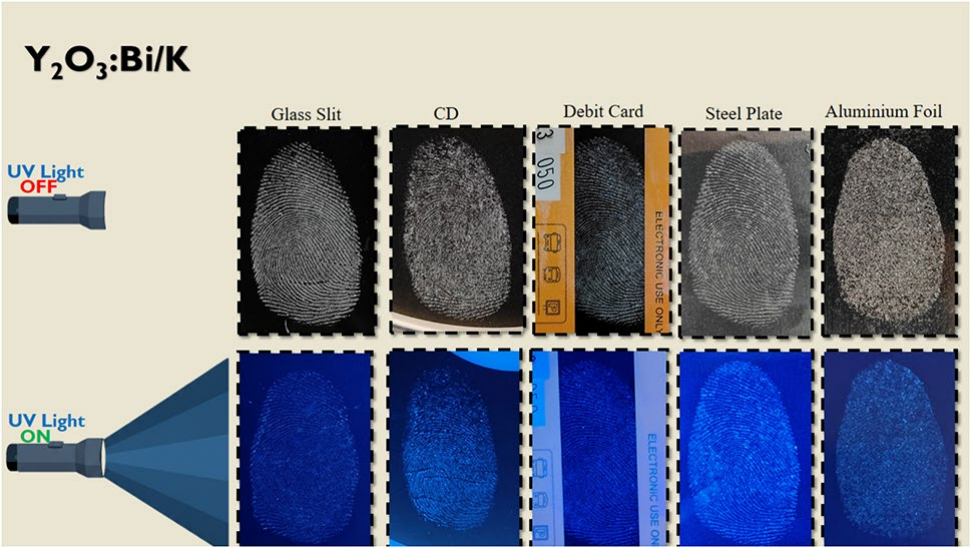
Fluorescence images of latent fingerprints stained by Y₂O₃: Bi/K phosphors.

**Fig. 38:**
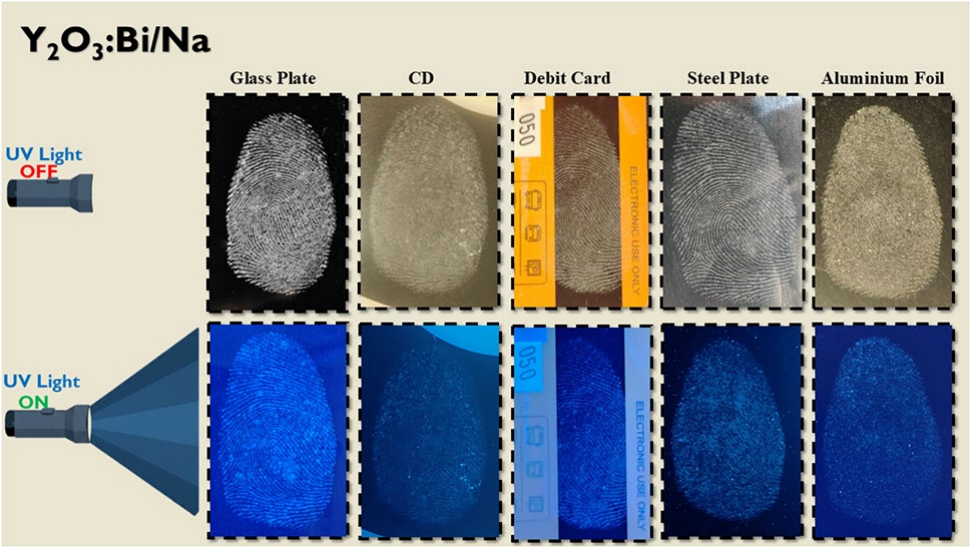
Fluorescence images of latent fingerprints stained by Y₂O₃: Bi/Na phosphors.

**Fig. 39:**
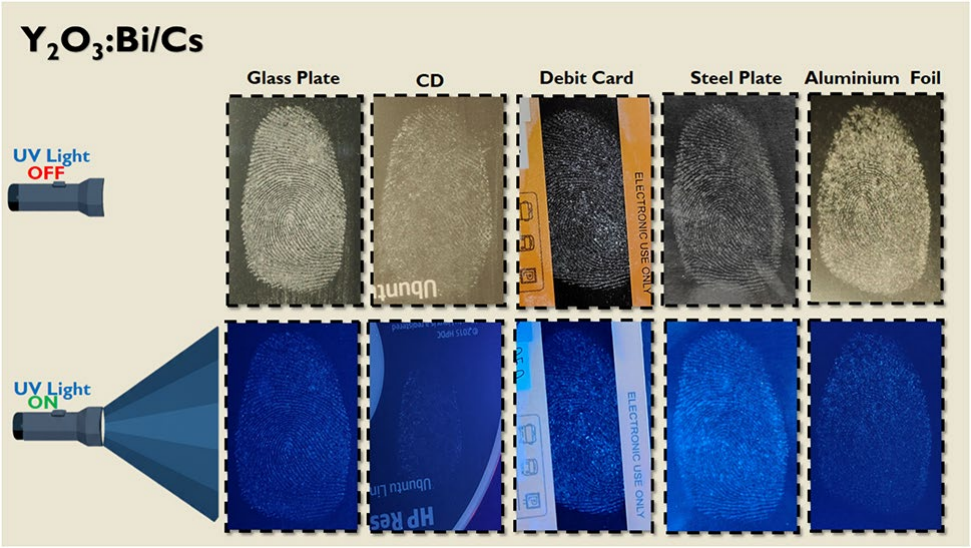
Fluorescence images of latent fingerprints stained by Y₂O₃: Bi/Cs phosphors.

In this study, we studied all Y₂O₃, Y₂O₃: Bi, Y₂O₃: Bi/Li, Y₂O₃: Bi/Na, Y₂O₃: Bi/K, and Y₂O₃: Bi/Cs prepared phosphors for fingerprint application. Concentration used for fingerprint application is Y₂O₃: Bi(0.3 mol%) , Y₂O₃: Bi(0.3 mol%)/Li (0.9 mol%), Y₂O₃: Bi(0.3 mol%)/Na(0.9 mol%), Y₂O₃: Bi(0.3 mol%)/K(0.9 mol%),and Y₂O₃: Bi(0.3 mol%)/Cs(0.6 mol%) because it has maximum emission intensity. We study the finger on different surface such as glass, aluminium foil, steel surface, CD, and debit card. Figures 35,36,37,38 and 39 show the fingerprint on different surfaces for Y₂O₃: Bi, Y₂O₃: Bi/Li, Y₂O₃: Bi/K, Y₂O₃: Bi/Na, and Y₂O₃: Bi/Cs respectively.

On comparing the latent fingerprints using all phosphors on all different surfaces. We found that Y₂O₃: Bi has the best result on surfaces like glass, aluminum, and steel. We found that phosphors such as Y₂O₃: Bi/Li have good latent fingerprint detection on surfaces like glass, CD, and steel. Phosphor such as Y₂O₃: Bi/Na has good detection of fingerprints on surfaces like debit cards and steel. Phosphor Y₂O₃: Bi/K can be used for surface like debit card and steel, while Y₂O₃: Bi/Cs can be useful for glass surfaces.

**Fig. 40:**
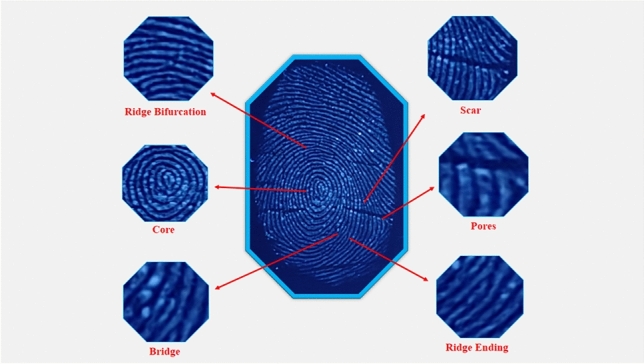
Latent fingerprint of Y₂O₃: Bi/Li on glass surface.

**Fig. 41:**
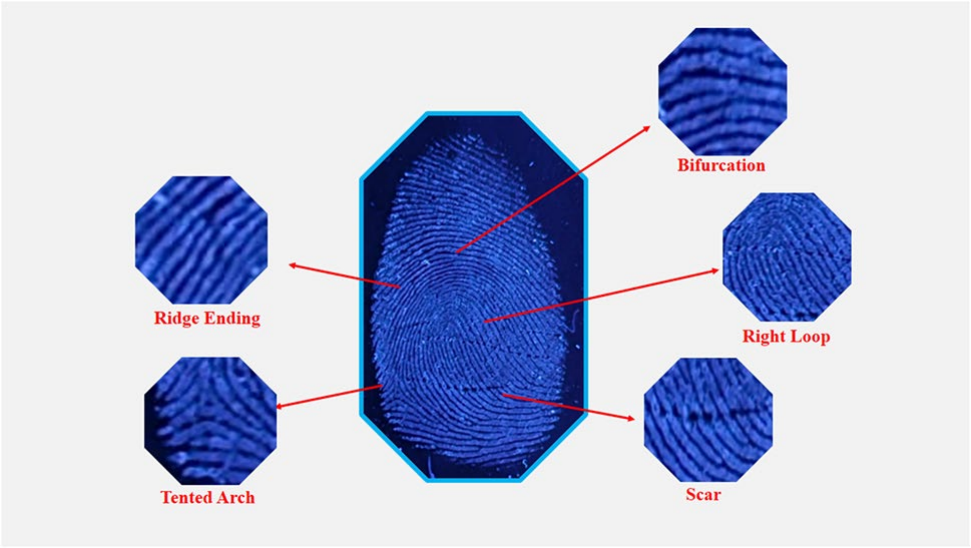
Latent fingerprint of Y₂O₃: Bi/K on glass surface.

Latent fingerprints produced on a glass surface using Y₂O₃: Bi/Li and Y₂O₃: Bi/K phosphors under UV light are visible in Figs. 40 and 41. The great contrast and high sensitivity of these phosphor materials toward surface trace residues are demonstrated by the clear viewing of fingerprint patterns. Ridge bifurcation, ridge ends, cores, bridges, scars, and pores are some of the intrinsic characteristics that make a fingerprint distinctive. In forensic analysis, accurately identifying these characteristics is crucial to proving a person’s uniqueness and legitimacy^42,43^. Different fingerprint characteristic regions are clearly visible, as illustrated in the Figs. 40 and 41. The fingerprint’s core features well-separated ridge lines, making it easy to tell how far apart successive ridges are from one another. The delicate structural elements are highlighted by the clear characterization of ridge bifurcation and ridge ends. Additionally, it is possible to identify tiny holes on the ridge surface and scars in between the ridges, which attests to the produced image’s great resolution and clarity. These findings imply that Y₂O₃: Bi/Li and Y₂O₃: Bi/K phosphors have high photoluminescent efficiency and surface adhesion, making latent fingerprints visible on smooth, nonporous surfaces like glass. Thus, the application of such luminous phosphors under UV-visible stimulation offers a highly dependable, non-destructive, and promising method for fingerprint detection that can be expanded to a variety of forensic and security applications.

## Conclusion

An efficient method to adjust the optical performance and functional application of Y₂O₃-based phosphors is to engineer the local symmetry environment of Bi^3+^ activator ions through alkali metal lattice modification. In this work, urea-assisted combustion was effectively used to create Bi-doped Y₂O₃ phosphors co-doped with Li^+^, Na^+^, K^+^, and Cs^+^ for latent fingerprint detection applications. A single-phase cubic crystal structure with space group (I –a 3) was confirmed by X-ray diffraction (XRD) research. Because of lattice contraction brought on by ionic substitution, the addition of dopant ions resulted in a reduction in crystallite size. To comprehend the causes of defect production and structural distortion, the change in lattice characteristics with various dopants was methodically examined. While FTIR spectra verified the existence of Y–O stretching vibrations, confirming the stability of the Y₂O₃ host lattice, SEM micrographs showed irregularly shaped particles with an average size of about 140 nm. Bi^3^⁺ ions have been shown by optical characterisation to inhabit two different symmetry sites in the cubic lattice: the non-centrosymmetric C₂ site and the centrosymmetric S_6_ site. Bi^3+^ showed distinct emission characteristics that corresponded to intra-configurational ^1^S₀ → ^3^P₁ and ^1^S₀ → ^1^P₁ transitions because of these two symmetry settings. Bluish-white emissions were created by the emission spectra excited at 329 and 337 nm, whereas a primarily blue emission was produced by excitation at 374 nm. Alkali ion co-doping greatly changed emission intensity but did not change the spectrum profile. The samples with the highest photoluminescence intensities under excitations of 329 nm, 337 nm, and 374 nm were Y₂O₃: Bi/K, Y₂O₃: Bi/Na, and Y₂O₃: Bi/Li. Interestingly, at 374 nm excitation, Y₂O₃: Bi (0.3 mol%)/Na (0.9 mol%) showed almost 100% color purity. Additionally, the produced phosphors showed exceptional latent fingerprint visualization capabilities. On glass, CD, and steel surfaces, Y₂O₃: Bi/Li demonstrated high contrast fingerprint detection; Y₂O₃: Bi/Na was successful on steel and debit cards; Y₂O₃: Bi/K shown great contrast on steel and debit cards; and Y₂O₃: Bi/Cs was especially well-suited for glass substrates. Bi and alkali co-doped Y₂O₃ phosphors appear to be interesting options for multicolor luminous materials and forensic fingerprint detection applications based on the combined structural and optical analysis.

## Supplementary Information

Supplementary Information 1.

Supplementary Information 2.

## Data Availability

All data supporting the findings of this study are available within the paper.
