# Supplementary material for: Lattice-engineered site symmetry control of Bi3⁺ activators for tunable luminescence and latent fingerprint detection
Source: Sci Rep. 2026 Apr 8;16:19005. doi: 10.1038/s41598-026-47106-4 (PMC13276067; doi:10.1038/s41598-026-47106-4)
Supplement: Supplementary file 1 — Supplementary Information 1. [file 41598_2026_47106_MOESM1_ESM.docx]

**Highlights**

1. **Local symmetry engineering:** Tunable optical performance of Y₂O₃:Bi³⁺ phosphors was achieved by modifying the local coordination environment of Bi³⁺ ions through alkali metal (Li⁺, Na⁺, K⁺, Cs⁺) lattice incorporation.
2. **Structural integrity and size control:** XRD confirmed a single-phase cubic Y₂O₃ structure (space group I2₁3), while alkali co-doping induced lattice contraction and reduced crystallite size (~140 nm).
3. **Dual-site Bi³⁺ emission behavior:** Optical analysis revealed that Bi³⁺ ions occupy both non-centrosymmetric (C₂) and centrosymmetric (S₆) sites, producing characteristic ¹S₀ → ³P₁ and ¹S₀ → ¹P₁ transitions with bluish-white to blue emission tunability.
4. **Enhanced photoluminescence efficiency:** Co-doping significantly improved emission intensity—Y₂O₃:Bi/K (329 nm), Y₂O₃:Bi/Na (337 nm), and Y₂O₃:Bi/Li (374 nm) exhibited the strongest luminescence, with Y₂O₃:Bi/Na showing nearly 100% color purity.
5. **Practical forensic application:** The synthesized phosphors enabled high-contrast latent fingerprint visualization on diverse surfaces, demonstrating material-specific substrate compatibility and potential for multicolor forensic imaging.
