# Supplementary material for: Lattice-engineered site symmetry control of Bi3⁺ activators for tunable luminescence and latent fingerprint detection
Source: Sci Rep. 2026 Apr 8;16:19005. doi: 10.1038/s41598-026-47106-4 (PMC13276067; doi:10.1038/s41598-026-47106-4)
Supplement: Supplementary file 2 — Supplementary Information 2. [file 41598_2026_47106_MOESM2_ESM.docx]

**Novelty**

The novelty of the present work lies in the systematic investigation of how the local site symmetry of Bi³⁺ ions (C₂ and S₆) within the cubic Y₂O₃ lattice influences their photoluminescence behavior. The study uniquely explores the role of alkali metal co-dopants (Li⁺, Na⁺, K⁺, and Cs⁺) as charge compensators and their impact on structural distortion, lattice contraction, and emission enhancement. Furthermore, the effect of three distinct Y³⁺ cation sites in the Y₂O₃ host on the optical transitions and crystal structure has been comprehensively analyzed to establish a clear structure–property correlation. Notably, this is the first report demonstrating the successful application of Bi and alkali co-doped Y₂O₃ phosphors for high-contrast latent fingerprint visualization on multiple substrates, highlighting their potential for forensic and photonic applications.
